# BswR controls bacterial motility and biofilm formation in *Pseudomonas aeruginosa *through modulation of the small RNA *rsmZ*

**DOI:** 10.1093/nar/gku106

**Published:** 2014-01-31

**Authors:** Chao Wang, Fuzhou Ye, Veerendra Kumar, Yong-Gui Gao, Lian-Hui Zhang

**Affiliations:** ^1^Institute of Molecular and Cell Biology, 61 Biopolis Drive, 138673 Singapore, ^2^School of Biological Sciences, Nanyang Technological University, 60 Nanyang Drive, 637551 Singapore and ^3^Guangdong Province Key Laboratory of Microbial Signals and Disease Control, South China Agricultural University, Guangzhou 510642, China

## Abstract

*Pseudomonas aeruginosa* relies on cell motility and ability to form biofilms to establish infections; however, the mechanism of regulation remains obscure. Here we report that BswR, a xenobiotic response element-type transcriptional regulator, plays a critical role in regulation of bacterial motility and biofilm formation in *P. aeruginosa*. Transcriptomic and biochemical analyses showed that BswR counteracts the repressor activity of MvaT, controls the transcription of small RNA *rsmZ* and regulates the biogenesis of bacterial flagella. The crystal structure of BswR was determined at 2.3 Å resolution; the monomer comprises a DNA-binding domain with a helix-turn-helix motif in the N terminus and two helices (α6 and α7) with a V-shaped arrangement in the C-terminus. In addition to the contacts between the parallel helices α5 of two monomers, the two helical extensions (α6 and α7) intertwine together to form a homodimer, which is the biological function unit. Based on the result of DNase I protection assay together with structural analysis of BswR homodimer, we proposed a BswR–DNA model, which suggests a molecular mechanism with which BswR could interact with DNA. Taken together, our results unveiled a novel regulatory mechanism, in which BswR controls the motility and biofilm formation of *P. aeruginosa *by modulating the transcription of small RNA *rsmZ*.

## INTRODUCTION

*Pseudomonas aeruginosa* is an important opportunistic human pathogen that causes acute or chronic infections in immunocompromised patients (1). The infectivity of *P. aeruginosa* is associated with its motility and biofilm formation (2), which is mutually exclusively switching between motile and sessile states on environmental conditions. Switching between motility and sessility aids the pathogen to survive harsh environmental conditions by increasing the efficiency of nutrient acquisition, escaping from toxic substances and accessing to favorable colonization sites (3,4). Mutants lacking motility and biofilm formation showed attenuated virulence in a burned mouse model (5), reduced invasion in corneal epithelial cells (6) and decreased adhesion to human respiratory mucin (7).

Swarming is one of the types of bacterial motilities through which bacterial cells move around to aid systemic infection and biofilm formation. It is a complex adaptation process in response to various environmental cues (8). In *P. aeruginosa*, the swarming motility is intimately associated with flagella assembly and type IV pilus (T4P) biogenesis (9). The assembly of flagella involves >50 genes that are coordinated by an array of regulatory proteins including the master regulator FleQ, the sigma factor RpoN, transcription factors FleR and FliA (10). Pilus biogenesis, an equally complicated process, also involves >40 genes and multiple signal transduction pathways (11). These signaling pathways include the two-component regulatory systems PilR/PilS and AlgR/FimS (12), the global regulator Crc (13), the virulence factor regulator Vfr (14) and the chemosensory systems encoded by the *pilGHIJK-chpABC *gene clusters (15,16).

In addition to these regulatory proteins, the small RNA regulatory system RsmA/RsmZ is also known to play critical roles in the regulation of bacterial motility and biofilm formation (17,18). RsmA, an RNA-binding protein, regulates swarming by positively modulating the biosynthesis of flagella and T4P (19,20). RsmZ, a noncoding regulatory small RNA, is an antagonist of the functional RsmA. Overexpression of RsmZ inactivates RsmA and abolishes bacterial swarming motility. Transcription of *rsmZ* is subject to the direct control of the GacS/GacA two-component system, and influenced by the sensor kinases RetS and LadS (21,22). Among them, it is the response regulator GacA that activates the *rsmZ* transcription by directly binding to the *rsmZ* promoter (17,23). Additionally, the H-NS–like protein MvaT acts as an *rsmZ* transcriptional repressor by binding to its promoter (23,24). Adding further to the complexity of the regulatory mechanisms that control motility and biofilm formation, evidence suggests the presence of some yet to be identified factors associated with the MvaT modulation of *rsmZ* expression (24).

Given the central role of RsmA/RsmZ system in modulation of *P. aeruginosa* cell motility and biofilm formation, it is of considerable interest to investigate the detailed regulatory mechanisms that govern its expression. Here we identified a novel transcription factor BswR, which is involved in the regulation of *P. aeruginosa* swarming motility and biofilm formation. Overexpression of *bswR* enhanced the transcription of *rsmZ*, downregulated the expression of flagellar and T4P genes and attenuated the bacterial swarming motility. We also found that BswR binds to the promoter of *rsmZ* and counteracts the repression of MvaT. Furthermore, we determined the crystal structure of BswR, which reveals that BswR forms a homodimer as the functional unit and provides clues on how BswR could interact with target promoter to regulate gene expression. These genetic and structural findings provide a new insight into the complicated and sophisticated regulatory mechanisms that govern the RsmZ/RsmA regulatory system and bacterial motility and biofilm formation.

## MATERIALS AND METHODS

### Bacterial strains, plasmids and culture conditions

Bacterial strains and plasmids used in this study were listed in Supplementary Table S1. Both *Escherichia coli* and *P. aeruginosa* strains were maintained in Luria–Bertani (LB) broth with shaking at 250 rpm or on LB agar plates at 37°C. When necessary, antibiotics were included in medium as follows: 150 and 300 mg·ml^−^^1^ carbenicillin, 5 and 50 mg·ml^−^^1^ gentamicin and 5 and 100 mg·ml^−^^1^ tetracycline for *E. coil* and *P. aeruginosa, *respectively.

Expression plasmid constructs were generated by standard methods and verified by DNA sequencing. The plasmids were transformed into *E. coli* by heat shock and *P. aeruginosa* strain by electroporation unless otherwise stated. To generate the *bswR*-overexpressing construct p19-bswR, the encoding region of *bswR* was amplified with polymerase chain reaction (PCR) primers 5′-GCGAATTCTGCTAGGTACCCGGCTAAAAG-3′ and 5′-GCGAATTCTCACAGTTCACTCCTTGTGC-3′, digested with EcoRI and inserted into the corresponding site of pUCP19. The p19-PA2781 was constructed similarly by using the PCR primers 5′-GCGAATTCTGAACAAAAGCTTGGATTCAG-3′ and 5′-GCGAATTCCTCAGCCTCGCGCCAG-3′. The *rsmZ* reporter construct pME-*rsmZ* was generated by cloning the EcoRI-digested *rsmZ* promoter into the enzyme site of pME6016 (25). PCR primers for pME-*rsmZ* construction were 5′-GGAATTCCAGTGACGGAAAACCTTAG-3′ and 5′-GGAATTCCCTGTACGCAGGAGTGATA-3′. To delete the 16th–99th amino acids of BswR, the construct pEX18G-bswR1 was prepared by overlapping PCR to generate the *bswR* allele *bswR1*, which lacks an internal fragment corresponding to nucleotides from 48th through 297th base pair (bp). The mutated allele was integrated into the chromosome of *P. aeruginosa* by homologous recombination, and the plasmid vector fragment was removed as previously described (26). The resultant mutants were verified by PCR and DNA sequencing.

### β-Galactosidase assays

β-Galactosidase activity was measured in duplicates and repeated at least twice. Bacterial start cultures were diluted (1:1000) in LB, grown for 8 h at 37°C and then aliquots were taken for measurement of enzyme activity. The β-galactosidase activity was quantified as previously described (27).

### Transposon mutagenesis

The mariner transposon carried by plasmid pBT20 was used for mutagenesis of *P. aeruginosa* PAO1 following the procedures as described (28). Mutants were screened on agar plates, which is a basic minimal nutrient medium supplemented with 0.2% mannitol as sole carbon source, 0.2% ammonia sulphate as sole nitrogen source and gentamicin (50 mg·ml^−^^1^) for selection of transposon mutants. The colonies showing smaller size than wild-type control were selected for further analysis. Arbitrary PCR, using primers of 5′-GTCGASWGANAWGNA-3′ with 5′-GTGCAAGCAGATACGGT GACGAT-3′ and 5′-TGACGATCCCGCAGTGGCTCTC-3′, was used to identify the genes disrupted by transposon insertion. The DNA sequences flanking the transposon insertion were analyzed with the NCBI BLAST server (http://www.ncbi.nlm.nih.gov/blast/) and the *Pseudomonas* genome database (http://www.pseudomonas.com/).

### Transcriptional profiling

PAO1, PAO1(p19), ΔbswR and ΔbswR(bswR) cultures were grown overnight in LB medium. Bacterial cells were then diluted to OD_600_ of 0.05 and grown at 37°C with shaking at 250 rpm. When OD_600_ reached ∼1.0, cells were harvested and total RNAs were extracted with RNeasy Protect Bacteria Mini kit (Qiagen). DNA contaminants were removed by DNase I. The quality of purified RNAs was analyzed by electrophoresis and quantified by Nanodrop 1000 Spectrophotometer (NanoDrop Technologies). Reverse transcription, fragmentation and complementary DNA labeling were conducted as described by the manufacturer (Affymetrix). The processed samples were hybridized to the Affymetrix GeneChip of *P. aeruginosa*, and chips were washed, scanned and analyzed following the instructions from the manufacturer. Hybridization signals were processed using the statistics software MAS-5.0 from Affymetrix.

### Quantitative real-time reverse transcription polymerase chain reaction

RNA samples were prepared as described above for transcriptional profiling analysis. An aliquot of 0.1 µg total RNAs was used as template, and real-time reverse transcription-PCR analysis was performed on Lightcycler v1.5 (Roche), using the SYBR®Green RT-PCR kit (Qiagen). The housekeeping gene *rpoC*, which encodes the β′ subunit of RNA polymerase, was included as an internal control. Results were presented as the ratio of target gene expression level versus the control gene (29). Real-time RT-PCR primers specific to *rsmZ* were 5′-CGTACAGGGAACACGCAAC-3′ and 5′-ATTACCCCGCCCACTCTTC-3′, and the primers for *rpoC* were 5′-CTGTTCAAGCC GTTCATTTTC-3′ and 5′-CTTGATGGTGGTGGCCATA-3′, respectively.

### Protein expression and preparation

For biochemical assays, protein expression constructs were generated using the vector pET28a. For preparation of BswR, the encoding region of *bswR* was amplified with PCR primers 5′-CGGGATCCCTAGGTACCCGGCTAAAAGC-3′ and 5′-CGGATGCGTGTCGCGTAG-3′, digested with BamHI and HindIII and cloned into the corresponding sites of pET28a (Novagen). For preparation of SpdH, the full-length *spdH* was amplified from the genomic DNA using the PCR primer pair 5′-CGGGATCCATGACCATCTCCCGCCGCGA-3′ and 5′-CCCAAGCTTCCGCTGCCGTCGCCGTCCT-3′, and cloned into pET28a in the same way as described for *bswR*. After verification by DNA sequencing, pET28a-bswR and pET28a-spdH were, respectively, transferred into *E. coli* BL21(DE3), and the transformants were grown in LB media containing 100 mg·ml^−^^1 ^kanamycin. Protein expression was induced by addition of 0.1 mM IPTG at 18°C for overnight. Cells were then harvested by centrifugation (4500 rpm, 30 min, 4°C), disrupted by French pressure cell, and the lysates were cleared by ultracentrifugation (25 000 rpm, 1 h, 4°C). For preparation of MvaT, the full-length *mvaT* was first cloned into pET28a using PCR primers 5′-GCGCATATGTCCCTGATCAACGAATATCG-3′ and 5′-GGGCTCGAGGCCGAGCAGGGTGGCCCAG-3′, and then the fragment carrying *mvaT* and His-tag was inserted into the pUCP19 vector to obtain p19-mvaT. After sequence verification, the vector p19-mvaT was then transferred into ΔmvaT(P*_rsmZ_*-lacZ) for protein purification. His_6_-tagged MvaT was then purified from the *P. aeruginosa* strain using Ni^2+^-affinity chromatography as recommended by Qiagen, followed by dialysis against the electrophoretic mobility shift assay (EMSA)-binding buffer (10 mM Tris, pH 7.5, 50 mM KCl, 1 mM dithiothreitol). Protein purity and integrity were examined by sodium dodecyl sulphate-polyacrylamide gel electrophoresis, and the concentration was determined by Bradford protein assay (Bio-Rad).

For crystallographic analysis, *bswR* was amplified by PCR and cloned into modified pET26b(+) vector (Novagen), in which BswR was expressed as a C-terminal 6×His-tagged fusion protein. The expression vector pET26M-bswR was then transformed into *E. coli* BL21(DE3) and expressed as described above. The BswR protein was first isolated by Ni-NTA column (GE healthcare) under a linear gradient elution of 0.0–0.5 M imidazole in the buffer 50 mM NaH_2_PO_4_, pH 7.5, 300 mM NaCl, 5 mM β-mercaptoethanol (β-ME), and further purified by HiLoad superdex 75 26/60 gel filtration column (GE healthcare), with the elution buffer 20 mM Tris, pH 7.5, 200 mM NaCl and 5 mM β-ME. The purified proteins were concentrated to 5.2 mg·ml^−^^1^ and stocked in the buffer (20 mM Tris pH 7.5, 200 mM NaCl, 5 mM β-ME) for crystallization trials.

### Electrophoretic mobility shift assay

The promoter region of *rsmZ* was labeled with biotin by PCR using primers 5′-Biotin-TTCCAGTGACGGAAAACCTTAG-3′ and 5′-Biotin-TTCCCTGTACGCAGGAGTGATA-3′. The control probe pexsC that extended the promoter region of *exsC *from *P. aeruginosa *PAO1 was PCR-amplified using 5′-biotinylated primers 5′- CTCCTAAAGCTCAGCGCATG-3′ and 5′-GTCAGTCCTATTTCACCCAGAG-3′. The biotinylated promoter was purified with QIAquick PCR Purification kit (Qiagen) and quantified by Nanodrop spectrophotometer. EMSA was carried out using LightShift® Chemiluminescent EMSA kit following the manufacturer’s instruction with modifications (Pierce). Each reaction (10 µl) contained the biotin-labeled probe (0.2 ng), 1 µg poly (dI•dC) (2'-deoxyinosinic-2'-deoxycytidylic acid) and various amounts of His_6_-BswR protein as indicated. In parallel, His_6_-SpdH was included in the assay as control. The reaction mixtures were incubated at 25°C for 20 min, and then the samples were separated on 6% polyacrylamide gels. The DNA probes were then transferred onto a nylon membrane, cross-linked by ultraviolet light at 254 nm and detected by chemiluminescence as described by the manufacturer (Pierce).

### Motility assay

The motility of *P. aeruginosa* was examined on LB agar (1.5%) plates at 37°C. Unless stated otherwise, 2 μl of fresh *P. aeruginosa *cultures was spotted in the center of plates and grown at 37°C for 2 days. Swarming, swimming and twitching motilities were respectively determined as described previously (4). Results were recorded after incubation overnight at 30°C.

### Biofilm assay

Biofilm formation was determined in 15-ml borosilicate tubes. Briefly, 1 ml of LB medium was inoculated to a final OD_600_ of 0.1 and statically incubated at 37°C for 6 h. Biofilm cell mass was virtualized by staining with 0.1% crystal violet and quantified by measuring the absorbance at 595 nm after solubilizing crystal violet in 96% ethanol. Each experiment was independently repeated for at least three times.

### DNase I protection assay

The promoter region of *rsmZ *was amplified by PCR with FAM-labeled primers 5′-(6-FAM)-ATTCTGGAGAAGAATGGCCTGTC-3′ and 5′-TTGCGTGTTCCCTGTACGC-3′ from the genomic DNA of *P. aerugionsa* PAO1. The experiment was done as described previously (30) in four repetitions with some modifications. Briefly, 100 ng of DNA was diluted in 20 µl of gel shift buffer, then 5 µl of purified BswR and 13 µl of water were added to the reaction; binding was allowed to proceed for 20 min at room temperature. Following several trials, 10 U of DNase I (Roche) was added and incubated for 5 min, and then the enzyme was heat-inactivated for 5 min at 95°C; DNA was purified by using Qiagen PCR kit and eluted on 25 µl of water for further analysis. Dideoxynucleotide-based sequencing was carried out using Thermo Sequenase Dye Primer Manual Cycle Sequencing kit (USB) according to the manufacturer’s instructions, and samples were analyzed by using the Applied BioSystems 3730xl DNA analyzer.

### Crystallization and structure determination

Crystallization trials were carried out using sitting-drop vapor diffusion method by Robot Phoenix (Art Robbins Instruments) at 20°C. The diffraction quality crystals of BswR were obtained in the drop by mixing protein sample and reservoir solution (0.1 M HEPES, pH 7.5, 20% w/v polyethylene glycol 10 000) with a volume ratio of 100 nl to 200 nl. For data collection, crystals were cryoprotected in reservoir solution supplemented with 20% glycerol, and flash-frozen in liquid nitrogen.

Diffraction data set was collected in Bruker AXS X8 Proteum X-ray system (wavelength = 1.54178 Å) (Bruker Axs Inc., USA). The data set was indexed, scaled and integrated with iMosflm (31). Using 3LFP [Protein Data Bank (PDB) code] as a search model, initial phases were obtained by molecular replacement using program Balbes in CCP4 suite (32), followed by model building with ARP/wARP (33). Model visualization and manual model building were carried out with COOT (34), and the refinement was done with CNS (35). The final model was examined using PROCHECK (36), crystallographic data and refinement statistics are listed in Table 1. Protein interface areas were calculated using PISA sever (37). Structural illustrations were generated using PyMol (DeLano Scientific).

**Table 1.** Summary of crystallographic data and refinement statistics

Data collection	
Space group	C2
Unit cell dimensions	
a,b,c (Å)	a = 63.0, b = 43.9, c = 35.0
α,β,γ (°)	α = 90, β = 115.2, γ = 90
Resolution (Å)	30.0–2.3 (2.42–2.30)
R_sym _(%)	8.5 (22.1)
I/σI	6.4 (2.9)
Completeness (%)	97.3 (83.5)
Refinement	
Resolution (Å)	30.0–2.3
Number of unique reflections	3818
R_work_/R_free_ (%)	22.9/28.6
No. of atoms	
Protein	803
Water	50
Average B factor (Å^2^)	
Main chain	26.8
Side chain	29.9
Waters	41.3
Rmsd	
Bond length (Å)	0.005
Bond angle (°)	0.978
Ramachandran plot	
Most favored regions (%)	94.5
Additional allowed regions (%)	4.4
Generously allowed regions (%)	1.1
Disallowed regions (%)	0.0

^a^R_sym_ = ∑|I_i_ – <I>|/|I_i_| where I_i_ is the intensity of the i^th^ measurement, and <I> is the mean intensity for that reflection.

^b^Reflections with I>σ was used in the refinement.

^c^R_work_ = |F_obs_ – F_calc_|/|F_obs_| where F_calc_ and F_obs_ are the calculated and observed structure factor amplitudes, respectively.

^d^R_free_ = as for R_work_, but for 9.4% of the total reflections chosen at random and omitted from refinement.

^e^Individual B-factor refinements were calculated.

## RESULTS

### BswR regulates *P. aeruginosa* motility and biofilm formation

To identify unknown regulatory factors associated with the motility of *P. aeruginosa*, a transposon mutant library was screened for alteration in colony size on LB agar plates. Of ∼2000 mutants screened, three mutants with colony size smaller than the wild-type control were selected and designated as Mot1, Mot2 and Mot3 (Supplementary Figure S1A). Sequence analysis of the three mutants showed that transposon was inserted in *PA2285 *(Mot1), *PA3966* (Mot2) and the intergenic region of *PA2779* and *PA2780 *(Mot3), respectively. Among them, PA2285 encodes the transcriptional factor AmrZ associated with the regulation of *P. aeruginosa* motility (38), and PA3966 encodes a hypothetic protein with unknown function, whereas *PA2779* and *PA2780* encode a hypothetic protein and a putative DNA-binding protein, respectively. The mutant Mot3, in which the transcriptional expression of *PA2780 *was significantly upregulated by transposon insertion (Supplementary Figure S2), was selected for further analysis in this study.

**Figure 1.**
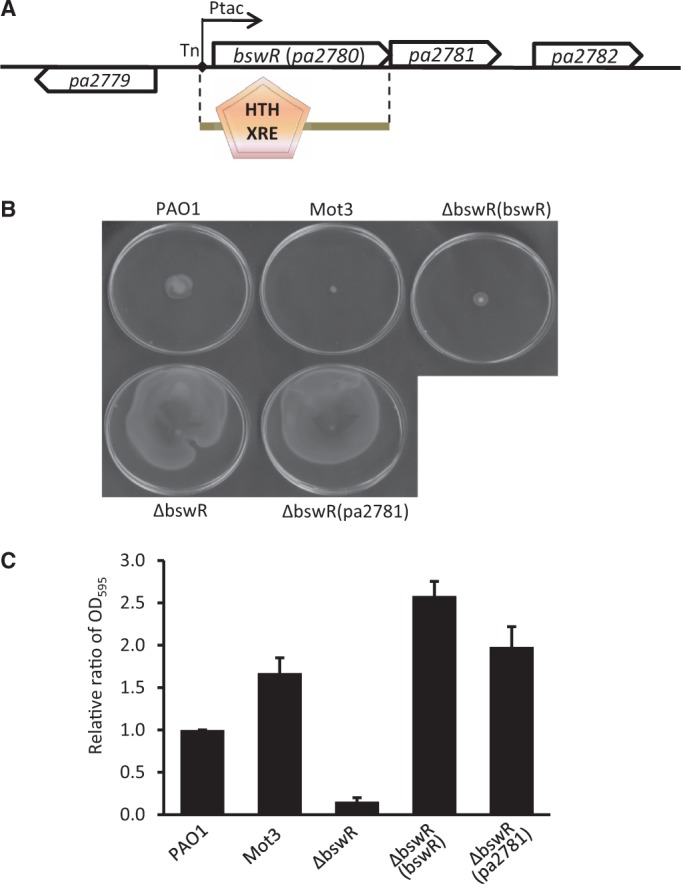
BswR modulates the motility and biofilm formation of *P. aeruginosa*. (**A**) Genetic organization of *bswR*. Arrow of ORF indicates the transcriptional direction. The solid diamond indicates the location of transposon insertion in the mutant Mot3 and the transcription direction of the transposon-borne P*_tac_* promoter is indicated by a solid arrow. The domain structure of BswR was predicted by using the SMART program (http://smart.embl-heidelberg.de/). (**B**) Representative swarming motility image of *P. aeruginosa *wild-type strain PAO1 and its derivatives. (**C**) Biofilm formation of PAO1 and its derivatives.

As shown in Figure 1A, the ORFs *PA2779* and *PA2780* were divergently orientated, sharing an intergenic region of 407 bp as putative promoters. The mariner transposon was inserted at the 12th bp upstream of *PA2780*, and the transposon-borne *tac* promoter was orientated in the same direction as that of *PA2780*, which is known to increase the transcriptional expression of downstream genes (28). Following *PA2780* is *PA2781*, a 339-bp ORF, which overlaps the last 1 bp of *PA2780* and possibly forms an operon with PA2780. Similar to *PA2780*, *PA2781* also encodes a hypothetic protein with unknown function. No discernible motifs were found in both proteins, but bioinformatics analysis suggested that PA2780, comprising 114 amino acid residues, possesses a potential xenobiotic response element (XRE)-type DNA-binding domain at its N-terminus (Figure 1A). According to its role in bacterial swarming regulation as described below, *PA2780* was designated hereafter as *bswR* (Bacterial SWarming Regulator).

In other bacterial species, the proteins containing the XRE-type DNA-binding domain have been implicated in regulating plasmid copy number and transcriptional expression of bacteriophage genes (39). However, the biological function of BswR in *P. aeruginosa* has not yet been characterized. To elucidate its role in bacterial motility, we generated the mutant ΔbswR by in-frame deletion of *bswR* in wild-type strain PAO1. We first checked the colony size on LB agar plates and found the deletion mutant formed much larger colonies than the wild-type and the *bswR*-overexpressing derivative (Supplementary Figure S1B), which was not due to altered bacterial growth rate (Supplementary Figure S3). We then examined the bacterial motility on specific agar plates for analysis of swarming, swimming and twitching motility (4). As shown in Figure 1B, we found deletion of *bswR* dramatically increased the bacterial swarming motility and the changed phenotype was rescued by *in trans* expression of the wild-type *bswR* but not *PA2781* in the deletion mutant ΔbswR (Figure 1B). We also tested the effect of *bswR* overexpression on bacterial swimming and twitching motility, but no significant change was observed (data not shown). These data suggest that BswR is specifically associated with the regulation of *P. aeruginosa *swarming motility.

Given that bacterial motility is critical for biofilm formation in *P. aeruginosa* (40), we tested the impact of *bswR* on biofilm formation. As depicted in Figure 1C, biofilm formation was almost completely abolished in the deletion mutant ΔbswR. In contrast, overexpression of BswR in the deletion mutant ΔbswR dramatically increased the biofilm formation. Surprisingly, overexpression of *PA2781 *in ΔbswR had no effect on bacterial swarming motility (Figure 1B), whereas this resultant strain produced more biofilm than that produced by the wild-type PAO1 (Figure 1C), suggesting that the hypothetical protein PA2781 may play a specific role in biofilm development, which is currently under investigation.

### BswR modulates the biosynthesis of flagella and type IV pili

**Figure 2.**
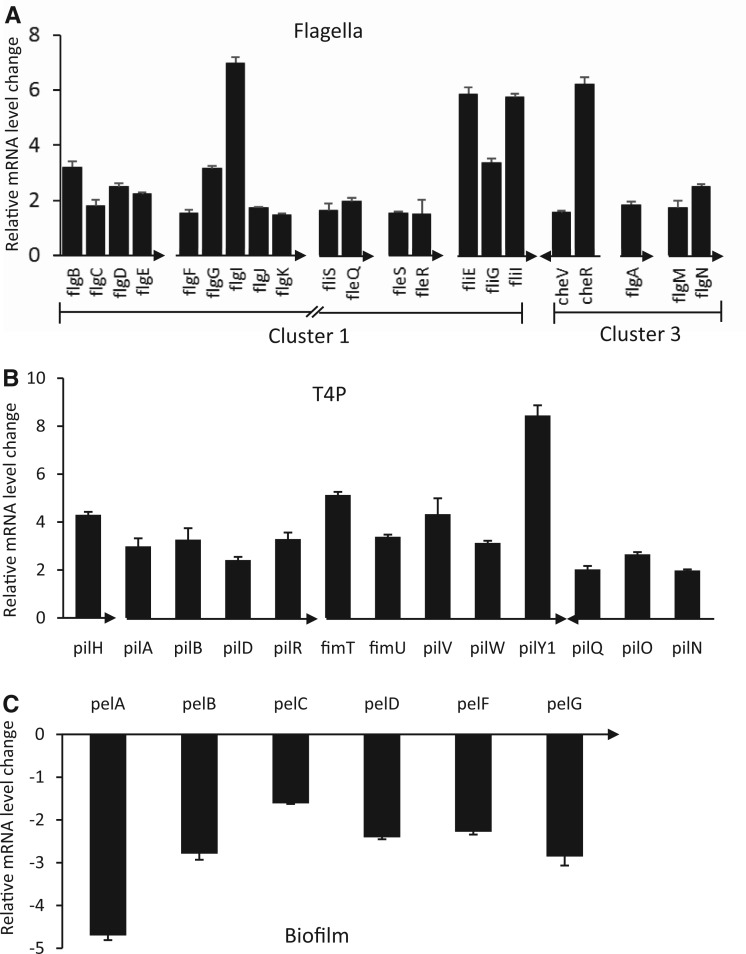
BswR regulates the transcription of the genes associated with the biogenesis of flagella (**A**), type IV pili (T4P) (**B**) and biofilm (**C**). Microarray analyses were performed using the exponential phase bacterial cells grown in LB medium. The BswR-regulated genes (≥2-fold) involved in the formation of flagella, T4P and biofilm were presented and organized according to their corresponding operons with arrows indicating the transcriptional direction. The transcriptional fold changes were presented for the genes increased in the mutant ΔbswR in comparison with wild-type strain PAO1.

To understand how BswR could regulate the swarming motility, we conducted microarray analysis to compare the global gene expression profiles of the deletion mutant ΔbswR and wild-type strain PAO1. The results showed that deletion of *bswR* significantly changed the expression patterns of 454 genes (≥2-fold), with 258 being upregulated and 196 downregulated (Supplementary Table S2). Considering the critical roles of flagella and T4P in bacterial motility and biofilm formation, we specifically compared the expression patterns of the genes associated with flagella and T4P in PAO1 and ΔbswR, and found that >40 flagella and T4P genes, including the gene clusters *flgB-flil*, *cheV-flgN*, *fimT-pilE* and pilQ-pilN, were upregulated (≥2-fold) in the deletion mutant (Figure 2A and B). These upregulation patterns were verified by real-time RT-PCR analysis (Supplementary Figure S4). Consistently, overexpression of *bswR* in the strain ΔbswR resulted in decreased (≥2-fold) transcriptional expression of 14 flagella and 15 T4P genes compared with the wild-type PAO1 (Supplementary Figures S2 and S5). These results suggest that BswR regulates swarming motility likely through modulation of flagella and T4P biogenesis.

In addition to flagella and T4P, extracellular polysaccharide (EPS) is another factor associated with biofilm formation in *P. aeruginosa* (41); we then analyzed the genes responsible for EPS production based on microarray data. Among the two gene clusters, i.e. the *pel* operon and the *psl* operon, known to be associated with EPS production, we found that only the expression of *pel* operon was significantly decreased in the ΔbswR mutant compared with the wild-type strain (Figure 2C). These data are in line with the previous study that mutation of the *pel *genes results in decreased biofilm formation (42) and establish a link between BswR and the *pel* genes in biofilm formation. Taken together, these results suggest that the influence of BswR on bacterial biofilm formation is not only related to its negative role in regulation of flagella and T4P biogenesis, but may also be associated with its positive effect on modulation of EPS production.

### BswR regulates the transcription of small RNA *rsmZ*

**Figure 3.**
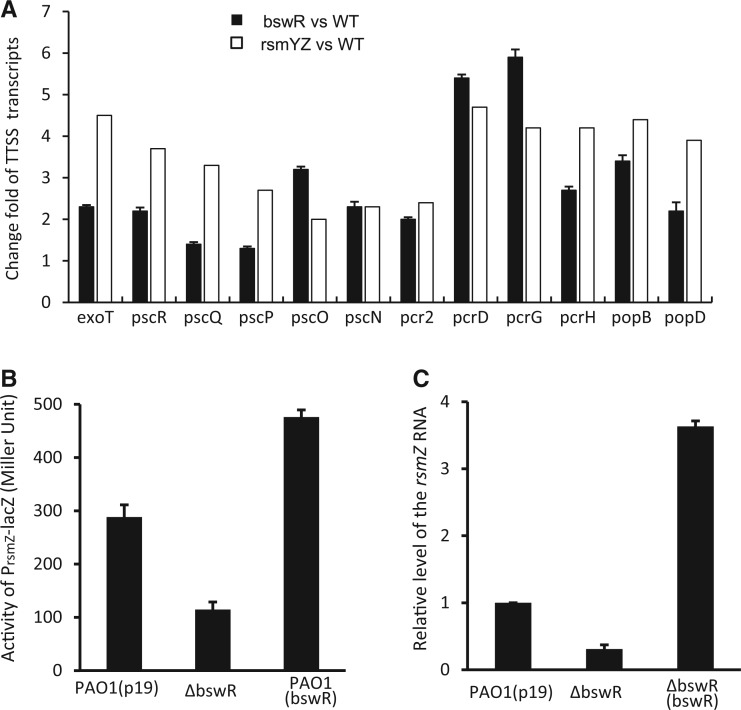
BswR regulates the transcription of small RNA *rsmZ*. (**A**) The gene expression patterns caused by *bswR* deletion were similar to those caused by *rsmZ*/*Y* deletion. Y-axis: the fold-changes of T3SS genes in ΔbswR compared with the wild-type and those in ΔrsmZ/Y as published previously (23). X-axis: Representatives of the TTSS genes whose mRNA levels were altered by deletion of bswR and *rsmY*/*Z* in *P. aeruginosa, *respectively. (**B**) Deletion of *bswR* resulted in decreased expression of *rsmZ*. The β-galactosidase activity conferred by P*rsmZ-lacZ *promoter fusion was determined when bacterial cells grown in LB medium reached at stationary phase. (**C**) The relative mRNA level of *rsmZ* revealed by real-time qRT-PCR analysis.

Besides the genes related to flagella, T4P and EPS biosynthesis, genes involved in protein secretion were also regulated by *bswR *(Supplementary Table S2). As shown in Figure 3A, expression of the genes for type III secretion system (T3SS) (including *exoT*, *exsE*, *pscRQPO*, *pcr1234, popB and pscEF*) was dramatically increased by deletion of *bswR*. Intriguingly, we noted that these expression patterns were mutually reciprocal with those caused by the deletion of the RNA-binding protein encoded by gene *rsmA* (20), and reminiscent to those caused by double deletion of small RNAs *rsmZ*/*Y *(Figure 3A) (23). It has been reported previously that deletion of *rsmZ*/*Y* results in increasing expression of T3SS genes and decreasing expression of T6SS genes, whereas deletion of *rsmA* increases the expression of T6SS genes but decreases the expression of T3SS and T4P genes (20,23).

Given that the RsmZ/*Y*-RsmA system is known to be associated with the regulation of *P. aeruginosa* motility (18), the above results motivated us to postulate that BswR might regulate the expression of *rsmZ*/*Y*. As no specific probes matching *rsmZ/Y* were designed in the GeneChips from Affimatrix, we tested this hypothesis by generating two reporter constructs by fusing the promoters of *rsmZ* (P*_rsmZ_*) and *rsmY* (P*_rsmY_*) separately to the coding region of *lacZ* and introduced these constructs into PAO1 and its derivatives. Analysis of the P*_rsmZ_*-directed β-galactosidase activity showed that deletion of *bswR* decreased the *rsmZ *expression, and overexpression of *bswR* increased the *rsmZ *expression level (Figure 3B). However, such differences were not observed when the P*_rsmY_*-lacZ fusion gene was assayed (data not shown). These results suggest that BswR regulates the transcriptional expression of *rsmZ *but not *rsmY*. Consistent with these results, real-time RT-PCR assay also showed that the transcript level of *rsmZ* was decreased by ∼60% in the *bswR*-deleted mutant and increased by ∼3-fold in the *bswR*-overexpression strain (Figure 3C).

### BswR requires GacA and MvaT for upregulation of *rsmZ*

**Figure 4.**
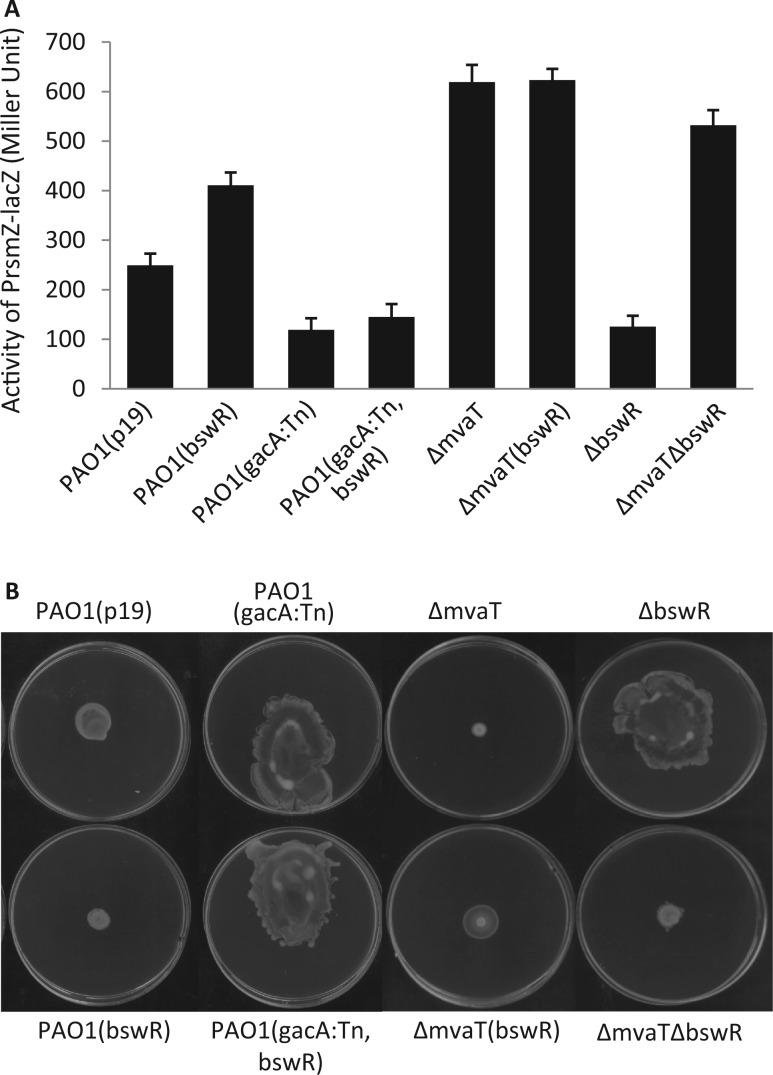
Regulation of *bswR* on bacterial motility is dependent on the RsmZ regulators GacA and MvaT. (**A**) The β-galactosidase activities of PAO1(P*rsmZ*-lacZ) and derivatives as specified. Bacterial cells were grown in LB medium, and the enzymatic activity was determined at stationary phase. (**B**) Representative images of swarming motilities of PAO1 and derivatives. Bacterial cells were inoculated at the center of the swarming plates and incubated at 30°C for 20 h before taking photographs.

GacA, a response regulator of the two-component system GasA/GacS in *P. aeruginosa*, has been well known to regulate *rsmZ* by directly binding to its promoter. It has been shown that disruption of GacA by transposon significantly reduced the *rsmZ* expression (23). To characterize the BswR-mediated regulation of *rsmZ*, we compared the transcription level of *rsmZ* in the wild-type strain and the relevant mutants. Consistent with previous report (23), disruption of *gacA *substantially reduced the transcriptional expression of *rsmZ* compared with the wild-type strain (Figure 4A). Notably, while *in trans *expression of *bswR* in wild-type strain increased the transcriptional expression of *rsmZ, *expression of *bswR* in the *gacA* mutant strain had no effect on *rsmZ* transcription (Figure 4A). The findings were further consolidated by examination of phenotype changes. As depicted in Figure 4B, overexpression of *bswR* inhibited the swarming motility of the wild-type PAO1. In contrast, the influence of overexpressed BswR was diminished when *gacA* was mutated, suggesting that BswR requires a functional GacA for the upregulation of *rsmZ* transcription.

In addition to GacA, the H-NS-like DNA-binding protein MvaT also binds to the *rsmZ* promoter and negatively regulates the transcription of *rsmZ *(24). To test whether BswR requires MvaT for the upregulation of *rsmZ*, we compared the transcription level of *rsmZ* in the wild-type and the relevant *mvaT* mutant strains. Consistent with the previous study (23), deletion of *mvaT* resulted in >2-fold increase in *rsmZ* transcription compared with the wild-type strain PAO1 (Figure 4A). In contrast, overexpression or deletion of the *bswR* gene in the background of *mvaT*-deletion mutant did not cause any change at the transcript level of *rsmZ* (Figure 4A). As a control, overexpression of *bswR* in PAO1 inhibited the swarming motility of the wild-type strain, and this inhibition was almost completely relieved in the *mvaT* mutant (Figure 4B). Given that overexpression of *bswR* increases the *rsmZ* transcription in the wild-type strain, these results suggest that BswR may act by counteracting the repressor MvaT in upregulation of *rsmZ*. Consistent with this notion, we found that the double deletion mutant ΔbswRΔmvaT had a comparable transcription level of *rsmZ* as that of the single deletion mutant ΔmvaT (Figure 4A). In line with these findings of genetic analysis, the swarming assay showed deletion of *bswR* in strain PAO1 increased the bacterial motility, but such an effect was vanished when *mvaT* was also deleted (Figure 4B).

### BswR binds to the promoter region of *rsmZ*

**Figure 5.**
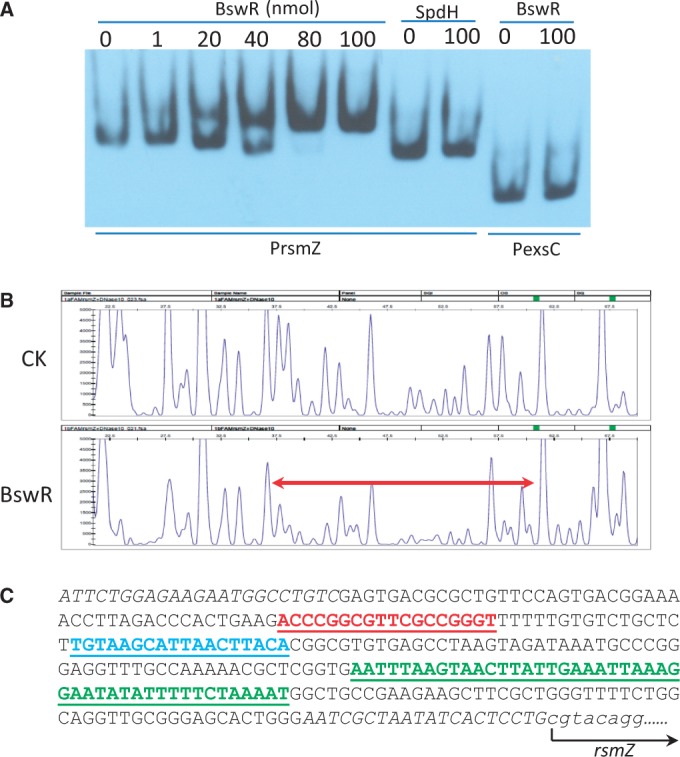
BswR binds to the promoter region of RsmZ. (**A**) EMSA of BswR binding to the promoter of *rsmZ*. EMSA assays were performed in presence of various amounts of His_6_-tagged BswR using the biotinylated promoter of *rsmZ* (P-*rsmZ*) as described in the text. His_6_-tagged SpdH, a protein associated with spermidine metabolism, and the biotinylated promoter of exsC (PexsC) from *P. aeruginosa *PAO1, were included as controls. (**B**) Identification of BswR binding sites in the promoter region of *rsmZ* using Dye Primer Sequencing on an automated capillary DNA analyzer. Top: electropherograms showing the protection region (red line) of the *rsmZ* promoter after digestion with DNase I following incubation in the presence (BswR) or absence (CK) of BswR. (**C**) Sequence analysis of the promoter region of *rsmZ*. Sequences for PCR primers were italicized. Binding sites for BswR as determined in this study were underlined in red; binding sites for GacA and MvaT were underlined in blue and green, respectively. The arrow indicated the transcription start site of *rsmZ*.

To characterize the function of BswR, we examined whether BswR could directly interact with the promoter of *rsmZ in vitro*. First, we expressed and purified the recombinant BswR as a His_6_-tagged protein and conducted the EMSA. As shown in Figure 5A, the mobility shift of the *rsmZ* promoter was affected by BswR in a dosage-dependent manner, demonstrating BswR binds to the *rsmZ* promoter. In contrast, the control protein SpdH, which is a spermindine hydrogenase in *P. aerugionsa *(43), did not interact with the *rsmZ* promoter. Furthermore, the purified BswR did not bind to the control DNA fragment PexsC, which is the promoter of gene *exsC* in *P. aeruginosa* (Figure 5A). Cumulatively, these results indicate that BswR positively regulates the transcriptional expression of the small RNA *rsmZ* by specifically binding to the promoter region of *rsmZ*.

To identify the binding sites of BswR in the *rsmZ* promoter region, we conducted the DNase I protection assay by using the PCR-amplified promoter region of *rsmZ*, which is labeled with the fluorescent dye FAM (fluorescein amidite). The results revealed that the DNA stretch ‘ACCCGGCGTTCGCCGGGT’ was clearly protected from the DNase I digestion on incubation together with BswR (Figure 5B). This protected region appeared as a palindrome, typical of the transcriptional factor binding sites in bacteria. In the promoter region of *rsmZ*, it extended from positions −226 to −208 relative to the transcriptional start site of *rsmZ*, upstream of the binding regions of GacA and MvaT (Figure 5C). Moreover, sequence analysis showed this protected region is absent in the promoter region of *rsmY*. This result was consistent with our findings that BswR has little effect on the transcription of *rsmY*, further supporting that BswR specifically regulates *rsmZ* by directly binding to its promoter region.

### Overall structure of monomeric BswR

To further understand the molecular mechanisms with which BswR interacts with the promoter of *rsmZ*, we proceeded with structural analysis by X-ray crystallography. The crystal structure of BswR was determined and refined to 2.3 Å resolution. While 101 residues were visible in the final model, the last 13 residues and the 6×His-tag in C-terminus were not well ordered in the electron density map and thus excluded. Then, the BswR protein model was refined and water molecules were located, finally giving R-work/R-free factor to 22.9/28.6%, respectively. The final model was examined using PROCHECK: 94.5% of the residues lie in the most favored regions, with the remaining 4.4 and 1.1% assigned to the additionally and generously allowed regions, respectively. The final refinement statistics for the structure are listed in Table 1.

**Figure 6.**
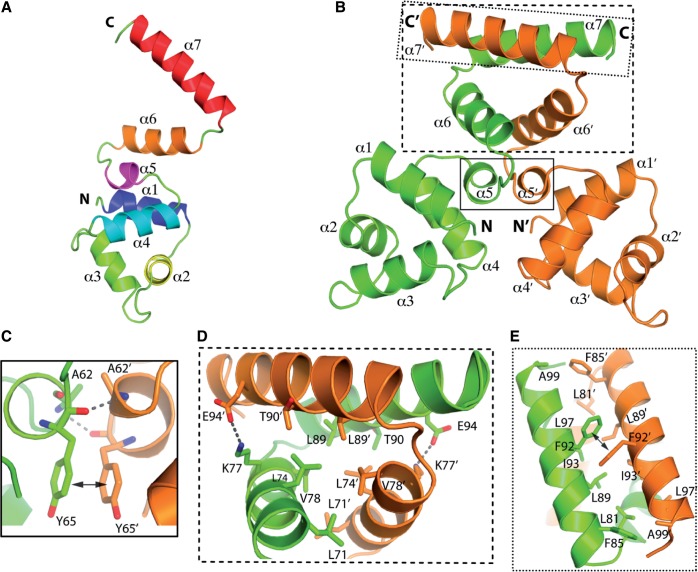
The structure of BswR and its dimerization. (**A**) Topology of the BswR monomer. The N- to C-termini of BswR is colored from blue to red. (**B**) The structure of BswR dimer. In a homodimer, one monomer is colored green, and the second brown (where the primes referred to the second monomer, same as below). The dimerization interface is framed by the black box, depicted as line, dashed line, and dotted line, respectively. (**C**) Enlarged view of dimer interaction along the parallel α5. The residues involved in dimerization interface are shown as stick model with labels. Hydrogen bonds between A62-Y65′ and A62′-Y65 were indicated as dashed lines, π- π stacking Y65-Y65′ was indicated with a double-ended arrow. (**D**) Enlarged view of dimer interface along the V-shaped terminal. Residues involved in dimerization were labeled and shown as stick model. Salt bridges (K77-E94′ and K77′-E94) are indicated as dashed lines. (**E**) Detailed interaction between α7 and α7′. Residues involved in dimerization are shown as stick model, π- π stacking F92-F92′ was indicated with a double-ended arrow.

The overall topology of BswR monomer contains seven helices, of which five form a compact helix bundle and the other two assemble as extended C-terminal helices (Figure 6A). Consistent with the prediction, our structure reveals that the N-terminus of BswR comprises α2-turn-α3, a typical helix-turn-helix (HTH) motif for DNA-binding (in which α2 is for stabilization and α3 for recognition). The two helices (α6 and α7) with a V-shaped arrangement in the C-terminus vary largely across different organisms, which meets the requirement for diverse ligand binding.

### Dimerization of BswR

Based on the gel filtration profile during protein purification, we found that BswR exists as a dimer in solution. This was further confirmed from the crystal structure: the two monomers with the V-shaped arrangement of the C-terminal helices intertwine together to form a homodimer by crystallographic symmetry. Using PISA and Contact (CCP4 suite) programs (33,39), we investigated the dimerization interface of BswR. In either monomer, 94 of 101 amino acids were exposed on the surface. On dimerization, an average interface area of 1438.7 Å^2^ was buried, accounting for 20.8% of the solvent-exposed surface area (6923.4 Å^2^) of each monomer. In total, 45 contacts were identified between two monomers with an intermolecular distance of <3.5 Å, the majority of which were located at the C-terminus. Furthermore, dimerization was mainly mediated through hydrophobic interaction, which involves the residues Leu71, Leu74, Leu81, Phe85, Leu89, Thr90, Ile93, Leu97 and Ala99 (Figure 6D and E). In addition, the intermolecular hydrogen bonds between the main chain N atom of Ala62 and the main chain O atom of Tyr65 (Ala62-Tyr65′, Tyr65-Ala62′, where the primes refer to the second monomer, same as below), the salt bridges (Lys77-Glu94, Glu94-Lys77′), as well as the aromatic π-π stacking interactions (Tyr65-Tyr65′ and Phe92-Phe92′) (Figure 6C and D), were found to contribute to the BswR dimerization. In the dimeric structure, BswR exhibits an uncommon large area of dimer interface, which is formed by the extension of C-terminal helical region (helices 5–7, residues 61–101). Clearly, the dimerization of BswR was mainly achieved by the interleaving of the two extended helices in its C-terminus, although the parallel helix 5 also contributes to the dimer formation.

XRE comprises the second-most frequently occurring regulator family in bacteria that control diverse biological functions, and these regulators share a characteristic N-terminal DNA-binding domain with a HTH motif, whereas the C-terminal region is extremely variable. Structural comparison of BswR with other XRE family proteins, restriction–modification (R–M) system controller proteins (C protein) Csp231I from *Citrobacter sp.* RFL231 (44), SinR from *Bacillus subtilis* (45), C protein Esp1396I from *Enterobacter sp.* RFL1396 (46) and EspR from *Mycobacterium tuberculosis* (47), shows that they share a similar fold for DNA-binding domain (Supplementary Figure S6), despite the variations mainly in the turn of HTH (α2-turn-α3) which could be of importance for orientating the recognition helix α3 for an optimal affinity for DNA (47). Except for C protein Esp1396I with one C-terminal helix, these proteins contain two C-terminal extended helices forming a V-shaped arrangement, which is involved in dimer assembling. The orientation of this V-shaped arrangement relative to the similar N-domain is varied, which consequently impacts on its DNA-binding (47). In contrast to the structural similarity, the sequence identity is relatively low (Supplementary Figure S6).

### Structural basis of BswR for DNA binding

**Figure 7.**
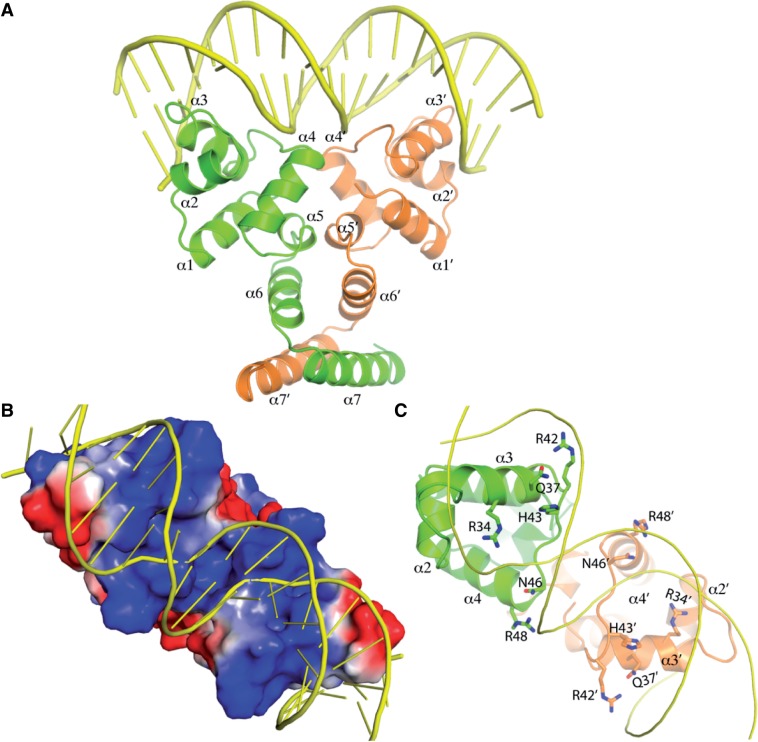
A proposed model of BswR interacting with DNA. (**A**) Model depicting BswR–DNA complex. Structural alignment of one protein dimer of Esp1396I–DNA complex (PDB ID: 3CLC) onto BswR dimer by fitting the two N-terminal domains was carried out with coot, subsequently the excess nucleotides far apart from BswR were removed. The dsDNA was shown as cartoon with sticks, same as (**B**). (B) A representative portion of the BswR–DNA complex structure with exposure of the HTH regions. Electrostatic potentials were rendered on the protein surface with a color scale between −40.0 *kT* e^−1 ^(Red) and +40.0 *kT* e^−1 ^(blue). The DNA was shown as cartoon with sticks. (**C**) The putative residues of BswR involved in DNA binding. The residues are predicted to interact with DNA are labeled and shown as sticks. For clarity, the DNA was shown as cartoon without sticks.

BswR possesses a calculated isoelectric point (pI) of 9.2, indicating that it is positively charged under physiological condition. EMSA assay showed that BswR can bind to the promoter region of *rsmZ*, and DNase I protection assay further revealed that ‘ACCCGGCGTTCGCCGGGT’, a palindromic sequence (underlined) in the *rsmZ* promoter, is the binding site of BswR. To elucidate how BswR binds to DNA, a structural alignment of BswR onto Esp1396I-DNA (PDB ID: 3CLC) resulted in a DNA-binding model (Figure 7A), in which no steric clash between BswR and the double-stranded DNA (dsDNA) was observed. In this model, the HTH motif in each monomer of BswR dimer is the major component to interact with the dsDNA, and the recognition helix α3 was inserted into the consecutive major groove of the dsDNA, in a canonical way to establish contact.

We also mapped the electrostatic surface of BswR dimer, as shown in Figure 7B. The strong positive charge was mainly located at the N-terminal DNA binding domain, and the remaining surface charges were evenly distributed. The dsDNA molecules were laid across the positive-neutral-positive patches in BswR dimer, and most positively charged region colored blue was covered, thereby demonstrating that the model is nicely consistent with the surface charge distribution. Compared with other XRE family transcriptional factors, several conserved residues were mapped out for BswR to bind DNA (Figure 7C). These residues, including Arg34, Gln37, Arg42, His43, Asn46 and Arg48 in the N-terminal domain, face toward the dsDNA and probably contribute to the direct contact with DNA.

## DISCUSSION

Bacterial motility and biofilm formation has been extensively investigated (3,8–11). In *P. aeruginosa*, swarming and biofilm formation are mutually exclusive, could be regulated by nitrogen limitation, controlled by quorum sensing and governed by a large number of transcriptional regulators (8,9). In this study, we discover a new layer of regulatory cascade that tailors swarming and biofilm formation in *P. aeruginosa*, by identification of a previously uncharacterized protein BswR (Figure 1). Genome-wide transcriptomic analyses show that BswR specifically controls the activity of flagella and T4P (Figure 2). Alteration of the *bswR* expression affected the swarming of *P. aeruginosa* and also affected the expression of genes involving the assembly of flagella and the biogenesis of T4P, indicating that BswR regulates bacterial swarming likely through compromising the function of flagella and T4P. However, mutation of *bswR* did not significantly influence the flagella-dependent swimming and the T4P-dependent twitching, suggesting that BswR have additional roles in regulation of bacterial swarming under these experimental conditions.

Our results provide new insights into how the small RNA *rsmZ* is regulated in response to cellular physiology and environmental stimuli (Supplementary Figure S7). RsmZ is a multifunctional regulator in *P. aeruginosa*. By counteracting the activity of RsmA, RsmZ acts as a negative regulator for bacterial swarming and a positive regulator for biofilm formation (18–20). In present study, we found overexpression of *bswR* inhibits bacterial swarming but favors biofilm formation, compatible with the previous results that overexpression of *rsmZ* resulted in loss of the swarming ability and increase of biofilm production (23). Identification of *rsmZ* as the target of BswR provides a molecular mechanism through which BswR regulates the bacterial swarming and biofilm formation.

Transcription of *rsmZ* is activated by GacA but repressed by MvaT, both physically binding to the promoter region of *rsmZ* (17,18,22,23,48). While the activity of GacA is titrated by a few two-component sensors, the repression of MvaT could be attenuated by some yet unidentified transcription factors (23). Our results provide a few lines of evidence suggesting that BswR is one of such factors. First, BswR activates the transcription of *rsmZ* (Figure 3). Second, BswR requires GacA for its activation (Figure 4). Third, this activation depends on the presence of MvaT (Figure 4). Fourth, BswR directly binds to the *rsmZ* promoter (Figure 5). Even so, the detailed mechanism through which BswR relieves the repression of MvaT requires further investigation. In the *rsmZ* promoter, binding site of GacA (TGTAAGCATTAACTTACA) is upstream of that of MvaT (AATTTAAGTAACTTATTGAAATTAAAGGAATATATTTTTCTAAAAT), separated by a DNA stretch with a length of 56 bp (Figure 5C). DNA footprint unravels that BswR binds to a separate site from that of MvaT, excluding the possibility that BswR and MvaT directly compete the binding sites for expression of RsmZ. In the crystal structure, BswR contains a positively charged DNA-recognition extension that dramatically deforms the promoter region during DNA binding (Figure 7), indicating that BswR may activate *rsmZ* by influencing the binding affinity of MvaT to the promoter region. However, previous results demonstrate that MvaT could associate with two small regulatory peptides for its repression activity (49), and moreover, MvaT functioning as a member of H-NS family protein usually causes DNA bending and thus facilitates distant protein–protein interactions (50). It is possible that BswR counteracts MvaT by directly binding to each other. In addition, the MvaT of *P. aeruginosa *has been reported to form both dimers and higher-order oligomers and the binding of MvaT to DNA targets depends on the oligomerization of MvaT dimers (50). Therefore, it is also possible that BswR counteracts MvaT by interfering with the protein–protein interactions between MvaT dimers.

Bacterial motility and biofilm are most important virulence determinants and survival strategies for microbial pathogens to cope with harsh environments and infecting hosts. Expression of these traits is under stringent regulation, and it responds to largely unidentified environmental signals. Although the regulatory cascade has been extensively studied, the environmental signals remain largely unknown. Structural comparison of BswR with other XRE family proteins reveals a similar fold for the N-terminal domain (Supplementary Figure S6). Further structural analyses suggest BswR functions as a homodimer, which uses positively charged helix to fit the major groove of the dsDNA for binding (Figure 7). In particular, BswR shares the most similar structure to C protein Csp231I, with a root-mean-square deviation (rmsd) of 1.169 Å for the main chain (Supplementary Figure S6). In *Citrobacter sp.* RFL231, Csp231I plays a key role in the temporal regulation of gene expression in bacterial R–M systems and are important mediators of horizontal gene transfer (44). The biological significance of this structural similarity is not known yet. Different from the N-terminal DNA-binding domain, the C-terminal region is structurally diversified with no typical homologs identified in the database. In the crystal structure, it comprises two alpha-helices with a V-shaped arrangement for dimerization. It is unknown whether this arrangement also involves the potential ligand binding for BswR, given that no ligand has been identified for these proteins, and little information is available on how the ligand-binding site could be formed. Analyses of previously published genome-wide microarray results show that the expression of BswR is not significantly influenced under the conditions of nutrient depletion, altered quorum sensing and increased temperatures, all of which have been implicated in bacterial motility and biofilm formation (51–54). Identifying the environmental signals that modulate the BswR expression and functionality will answer whether BswR represents a previously unappreciated signal pathway regulating the virulence and survival capability of *P. aeruginosa*.

## ACESSION NUMBERS

Atomic coordinate and structure factor of BswR have been deposited in PDB with accession code 4O8B.

## SUPPLEMENTARY DATA

Supplementary Data are available at NAR Online.

## FUNDING

Biomedical Research Council, A*STAR Singapore; Singapore National Research Foundation [NRF-RF2009-RF001-267 to Y.G.G.] and NTU-Startup (to Y.G.G.). Funding for open access charge: Agency for Science, Technology & Research, Singapore.

*Conflict of interest statement*. None declared.

## Supplementary Material

Supplementary Data
