# Supplementary material for: BswR controls bacterial motility and biofilm formation in Pseudomonas aeruginosa through modulation of the small RNA rsmZ
Source: Nucleic Acids Res. 2014 Jan 31;42(7):4563–76. doi: 10.1093/nar/gku106 (PMC3985676; doi:10.1093/nar/gku106)
Supplement: Supplementary Data [file supp_42_7_4563__index.html]

BswR controls bacterial motility and biofilm formation in Pseudomonas aeruginosa through modulation of the small RNA rsmZ — BswR controls bacterial motility and biofilm formation in Pseudomonas aeruginosa through modulation of the small RNA rsmZ — Supplementary Data 

# BswR controls bacterial motility and biofilm formation in *Pseudomonas aeruginosa* through modulation of the small RNA *rsmZ*

## Supplementary Data

files

**Files in this Data Supplement:**

- Supplementary Data - pdf file
