# Supplementary material for: BswR controls bacterial motility and biofilm formation in Pseudomonas aeruginosa through modulation of the small RNA rsmZ
Source: Nucleic Acids Res. 2014 Jan 31;42(7):4563–76. doi: 10.1093/nar/gku106 (PMC3985676; doi:10.1093/nar/gku106)
Supplement: Supplementary Data [file supp_gku106_nar-03509-v-2013-File003.pdf]

Supplementary Figure S1

A

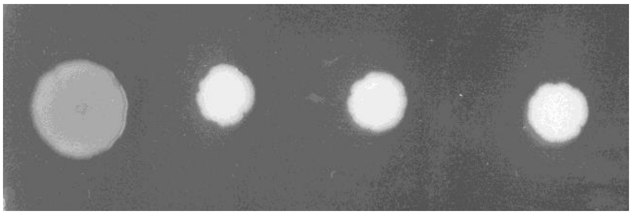

B

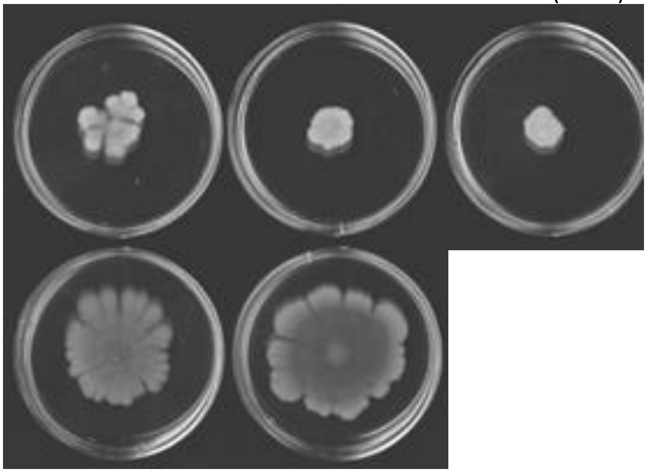

Supplementary Figure S1. BswR regulates colony sizes of *P. aeruginosa* on LB agar plates. A) Colony sizes of PAO1, Mot1, Mot2 and Mot3 on LB agar plates. B) Colony sizes of BswR-deviated mutants on LB agar plates.

Supplementary Figure S2

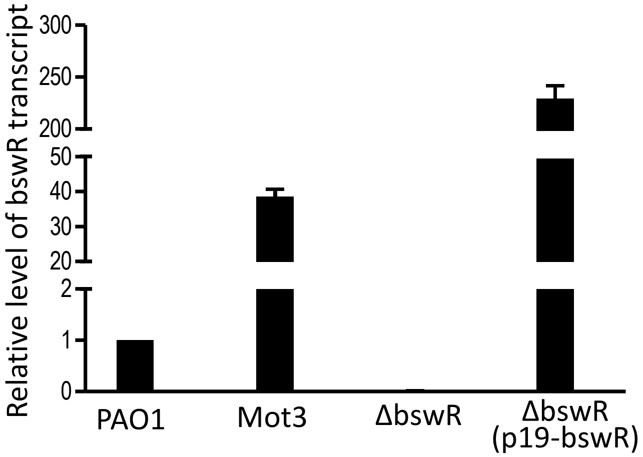

Supplementary Figure S2. Real time RT-PCR analysis of BswR transcript levels in the given bacterial strains. Total RNAs were isolated from bacterial cells at exponential phases grown in LB media at 37◦C with a shaking of 250 rpm. RT-PCR primers specific to BswR were 5'-ATGCGGACTTCCGAACAG-3' and 5'-CCTCGGGGCAATAGAGGTAG-3'. Relative transcript levels were normalized against rpoC, and the bswR transcript was arbitrarily set as 1.

Supplementary Figure S3

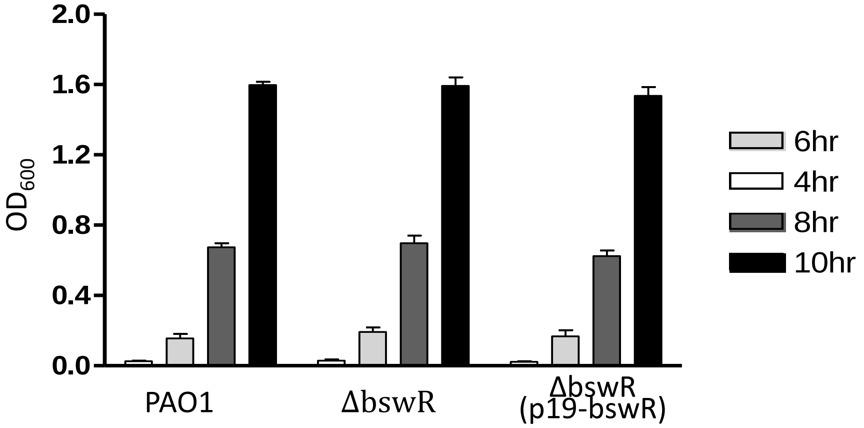

Supplementary Figure S3. Growth curves of BswR-related mutants in LB media. Fresh bacterial strains were inoculated in LB media with initial OD_{600} of 0.01 and grown at 37◦C with a shaking of 250 rpm. OD_{600} was measured at specified time points.

Supplementary Figure S4

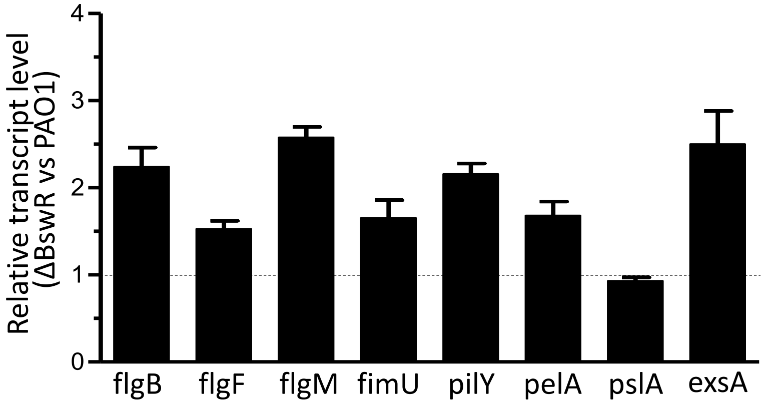

Supplementary figure S4. Real time RT-PCR analysis of transcripts of interest in the ΔbswR mutant compared to the wild type PAO1. Total RNAs were isolated from bacterial cells at exponential phases grown in LB media at 37◦C with a shaking of 250 rpm. Relative transcript levels were all normalized against the wild type strain with the internal control rpoC. Specific RT-PCR primers were listed as follows:

flgB: 5’- ccgagcagaaggacaagg-3’ and 5’- tcgacagtgttctggtcgat-3’;
flgF: 5’- aagatgctgtacgtctccatga-3’ and 5’- gagatgttcgccaggttgtt-3’;
flgM: 5’- taaggttgcccggatcaag-3’ and 5’- gattcgaagtcgagcagctt-3’;
fimU: 5’- accctgatcgagttgctgat-3’ and 5’- tgcttgaagttcggaatgg-3’;
pilY1: 5’- atgctacacccgccgtta-3’ and 5’- acgaataccagttggcgaag-3’;
pelA: 5’- gctgaagggtcacctgga-3’ and 5’- gtagtcgatggcgacgatg-3’;
pslA: 5’- gggtcccggagaactacaac-3’ and 5’- agcttctccaggtccttggt-3’;
exsA: 5’-cgagcggagaatcctctatg-3’ and 5’-cgatgtcgacgatgctca-3’;
rpoC: 5’-ctgttcaagccgttcattttc-3’ and 5’-cttgatggtggtggccata-3’.

Supplementary Figure S5

A

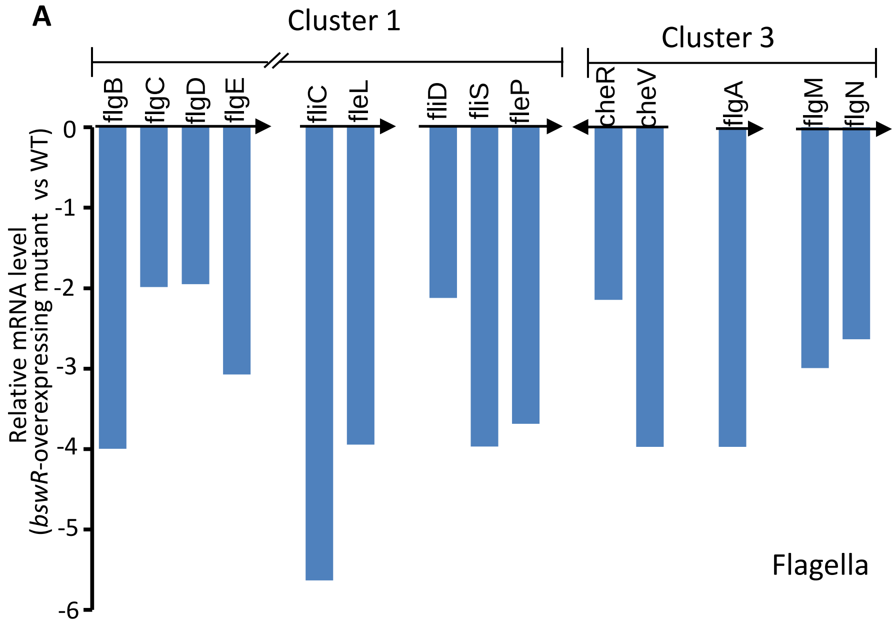

B

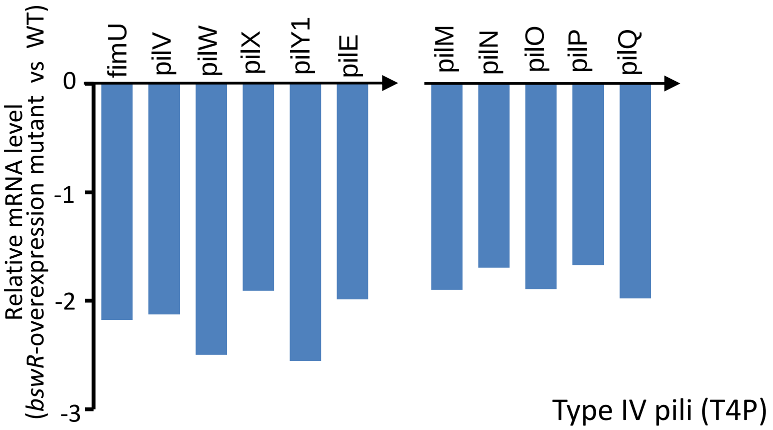

Supplementary Figure S5. Overexpression of BswR decreased transcription of genes for flagellar (A) and type IV pili (B). Microarray analyses were performed for bacterial cells grown in LB media. Representative genes were selected based on operons. Transcription direction of operons was indicated by arrows and relative location of operons was shown on the top. The transcriptional fold changes were presented for genes decreased in the BswR-overexpressing mutant PAO1(ΔBswR, p19-BswR) in comparison with the wild type strain PAO1 (WT).

Supplementary Figure S6

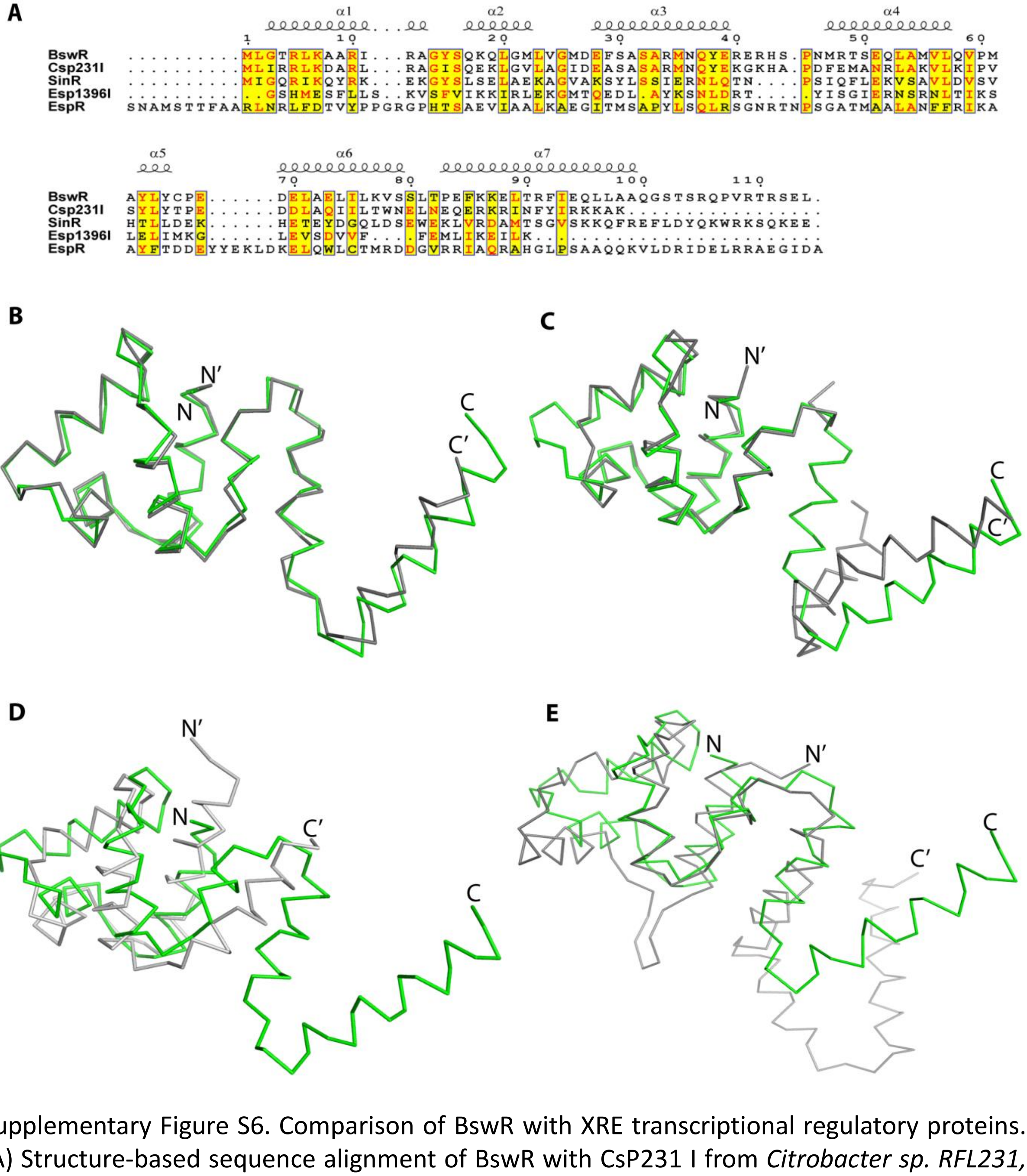

Supplementary Figure S6. Comparison of BswR with XRE transcriptional regulatory proteins. (A) Structure-based sequence alignment of BswR with CsP231 I from *Citrobacter sp. RFL231,* SinR from *Bacillus subtilis presented using ESPript*. C protein Esp13961 from *Enterobacter sp. RFL1396,* and EspR from *Mycobacterium tuberculosis*. Labels show the secondary structure of BswR. Identical residues are highlighted red and similar residues are framed in blue. (B) Superposition of BswR structure (colored green, same as below) with CsP231I (colored grey, PDB code 3LFP). (C) Superposition of BswR structure with SinR (colored grey, PDB code 1B0N). (D) Superposition of BswR structure with Esp13961 (colored grey, PDB code 3CLC). (E) Superposition of BswR structure with EspR (colored grey, PDB code 3QF3).

Supplementary Figure S7

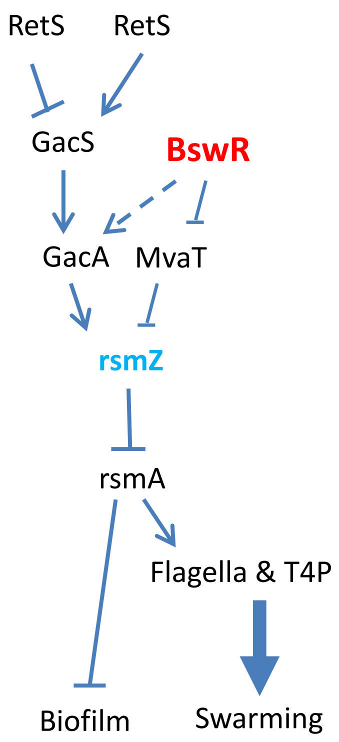

Supplementary Figure S7. Schematic presentation for the regulatory inputs of GacA, BswR and MvaT on the *rsmZ* expression and the outputs of flagella, type IV pili (T4P), swarming and biofilm formation. By directly binds to the promoter of small RNA *rsmZ*, BswR enhances its expression by counteracting the repression of MvaT and/or enhancing the activation of GacA. Once expressed, *rsmZ* antagonizes the activity of RsmA and thereby inhibits the function of flagella and T4P. Arrows indicate positive regulation and blocked lines indicate negative regulation.

Supplementary Table S1. Strains and plasmids used in this study*

Strains or plasmid	Description	Source or reference
E. coli		
DH5α	F– f80 d*lacZ*DM15 *endA1hsdR17* (r_k^- m_k^-) *supE44 thi-1 gyrA96* D(*lacZYA-argF*), used for plasmid transformation	Gibco
BL21(DE3)	*F⁻ ompT hsdS(rB⁻ mB⁻) dcm⁺ Tetʳ gal (DE3) endA Hte,* used for overexpressing 6XHis-tagged PA2780 and 6XHis-tagged SpdH	Novagen
SM10(pBT20)	Harboring mariner transposon for *P. aeruginosa* mutagenesis, Gm^r & Cb^r	(28)
**Plasmids**		
Tn7T-Gm-lacZ	mini-Tn7T cloning and delivery vector for the *lacZ* transcriptional fusion vector, Gm^r	(55)
Tn7-bswR	mini-Tn7T-Gm-lacZ carrying the promoter region of *bswR* (PA2780) for reporting its transcription	This study
pUCP19	Shuttle vector for gene expression in Pseudomonas	(56)
p19-bswR	pUCP19 carrying encoding region of *bswR* for overexpression of *bswR* in *P. aeruginosa*, Cb^r	This study
p19-2781	pUCP19 carrying encoding region of PA2781 for overexpression of PA2781 in *P. aeruginosa*, Cb^r	This study
p19-mvaT	pUCP19 carrying encoding region of mvaT and 6xHis tag for overexpression of mvaT in P. aeruginosa, Cbr	This study
pME-rsmZ	pME6016 carrying the promoter region of *rsmZ* for reporting the *rsmZ* transcription, Tc^r	This study
pEX18Gm	Suicide plasmid carrying *sacBR*, Gm^r	(57)
pEX18G-bswR1	pEX18Gm containing the *bswR* flanking region with the part of *bswR* being deleted in frame, Gm^r	This study
pET28a-bswR	pET28a carrying the encoding region of *bswR* for overexpression of His_6-tagged BswR in *E. coli*, Kn^r	This study
pET28a-spdH	pET28a carrying the encoding region of *spdH* for overexpression of His_6-tagged SpdH in *E. coli*, Kn^r	This study

P. aeruginosa

PAO1	Prototrophic laboratory strain, wild type strain	Laboratory collection
PAO1(p19)	PAO1 carrying the empty vector pUCP19, Cb^r	This study
Mot3	PAO1 carrying mariner transposon in the promoter of *pa2780* with motility-deficient phenotype, Gm^r	This study
ΔbswR	PAO1 with inframe deletion of *bswR*	This study
ΔbswR(bswR)	ΔbswR carrying the p19-bswR vector for overexpression of *bswR*, Cb^r	This study
ΔbswR(pa2781)	ΔbswR carrying the p19-2781 vector for overexpression of PA2781, Cb^r	This study
PAO1(P_{rsmZ}-lacZ)	PAO1 carrying the pME-*rsmZ* plasmid for reporting the *rsmZ* transcription, Tc^r	This study
ΔbswR(P_{rsmZ}-lacZ)	ΔbswR carrying the pME-*rsmZ* plasmid for reporting the *rsmZ* transcription, Tc^r	This study
PAO1(bswR, P_{rsmZ}-lacZ)	PAO1carrying the p19-bswR & pME-*rsmZ* vectors for overexpressing *bswR* and reporting the *rsmZ* transcription, Tc^r & Cb^r	This study
PAO1(bswR, P_{rsmZ}-lacZ, gacA:Tn)	PAO1 with a mariner transposon insertion destroying *gacA* and carrying the p19-2780 & pME-rsmZ vectors for overexpressing *bswR* and reporting *rsmZ* transcription, Tc^r &Cb^r & Gm^r	This study
ΔmvaT(P_{rsmZ}-lacZ)	PAO1 with deletion of *mvaT* carrying the pME-rsmZ plasmid for reporting the *rsmZ* transcription, Tc^r	This study
ΔmvaT(bswR, P_{rsmZ}-lacZ)	ΔmvaT(pME-*rsmZ*) carrying the p19-2780 & pME-rsmZ vectors for overexpressing *bswR* and reporting *rsmZ* transcription, Tc^r & Cb^r	This study
ΔmvaTΔbswR (P_{rsmZ}-lacZ)	PAO1 with double deletion of *mvaT* & *bswR* carrying the pME-rsmZ vector for reporting *rsmZ* transcription, Tc^r	This study
ΔmvaT(P_{rsmZ}-lacZ, p19-mvaT)	PAO1 with deletion of mvaT carrying the plasmids pME-rsmZ and the p19-mvaT, Cb^r, Tc^r	This study

* Symbol: Gm^r, gentamicin resistant; Cb^r, carbenicillin resistant; Tc^r, tetracycline resistant; Kn^r, kanamycin resistant.

REFERENCE

28. Kulasekara, H.D., Ventre, I., Kulasekara, B.R., Lazdunski, A., Filloux, A. and Lory, S. (2005) A novel two-component system controls the expression of Pseudomonas aeruginosa fimbrial cup genes. *Mol Microbiol*, **55**, 368-380.

55. Choi, K.H. and Schweizer, H.P. (2006) mini-Tn7 insertion in bacteria with single attTn7 sites: example Pseudomonas aeruginosa. *Nat Protoc*, **1**, 153-161.

56. Schweizer, H.P. (1991) Escherichia-Pseudomonas shuttle vectors derived from pUC18/19. *Gene*, **97**, 109-121.

57. Hoang, T.T., Karkhoff-Schweizer, R.R., Kutchma, A.J. and Schweizer, H.P. (1998) A broad-host-range Flp-FRT recombination system for site-specific excision of chromosomally-located DNA sequences: application for isolation of unmarked Pseudomonas aeruginosa mutants. *Gene*, **212**, 77-86.

Supplementary Table S2. Genes with a minimum twofold change at transcript level in ΔbswR compared to the *Pseudomonas aeruginosa* PAO1 wild-type.

Positive values represent genes increased in expression in the ΔbswR mutant compared to PAO1 wild-type; negative values represent genes decreased in expression in the ΔbswR mutant compared to PAO1 wild-type. PA numbers, annotations and gene classes are from the Pseudomonas website (http://www.pseudomonas.com).

Gene name	Change fold (ΔBswR/wt)	Gene description
**Regulator**		
PA0149	3.1	probable sigma-70 factor, ECF subfamily
PA0151	2.3	probable TonB-dependent receptor
PA0612	-6.3	repressor, PtrB
PA0674	-4.2	VreA
PA0675	-2.6	ECF sigma factor, VreI
PA0676	5.3	sigma factor regulator, VreR
PA1097_fleQ	2	transcriptional regulator FleQ
PA1315	11.2	probable transcriptional regulator
PA1322	2.1	probable TonB-dependent receptor
PA1328	-3.4	probable transcriptional regulator
PA1347	-3.4	probable transcriptional regulator
PA1399	2.1	probable transcriptional regulator
PA1430_lasR	-2.2	transcriptional regulator LasR
PA1636_kdpD	3.8	two-component sensor KdpD
PA1637_kdpE	3.2	two-component response regulator KdpE
PA1707_pcrH	5.4	regulatory protein PcrH
PA1713_exsA	2.3	transcriptional regulator ExsA
PA1760	-2.3	probable transcriptional regulator
PA1864	-20.1	probable transcriptional regulator
PA1898	2.1	quorum-sensing control repressor
PA1911	2	sigma factor regulator, FemR
PA1980	-4.5	response regulator EraR
PA2050	4.6	probable sigma-70 factor, ECF subfamily
PA2093	-2	probable sigma-70 factor, ECF subfamily
PA2227	4	AraC-type transcriptional regulator VqsM
PA2258_ptxR	3.1	transcriptional regulator PtxR
PA2320_gntR	-2.2	transcriptional regulator GntR
PA2335	10.1	probable TonB-dependent receptor
PA2467	3.5	Anti-sigma factor FoxR

Gene name	Change fold (ΔBswR/wt)	Gene description
PA2511	-14.8	probable transcriptional regulator
PA2519_xylS	-5.1	transcriptional regulator XylS
PA2588	-2.1	probable transcriptional regulator
PA2591	2.4	probable transcriptional regulator
PA2663	-2.6	psl and pyoverdine operon regulator, PpyR
PA2687_pfeS	2	two-component sensor PfeS
PA2810	-5.7	two-component sensor, CopS
PA2818	-3.4	aminoglycoside response regulator
PA3006	-2	transcriptional regulator PsrA
PA3206	-2.3	probable two-component sensor
PA3346	7.6	two-component response regulator
PA3410	2.5	probable sigma-70 factor, ECF subfamily
PA3462	-4.4	probable sensor/response regulator hybrid
PA3739	4	probable sodium/hydrogen antiporter
PA3885	4.2	protein tyrosine phosphatase TpbA
PA3932	15.1	probable transcriptional regulator
PA3946	2.5	Two-component sensor RocS1
PA3995	6.6	probable transcriptional regulator
PA4036	11.7	probable two-component sensor
PA4168	21.5	second ferric pyoverdine receptor FpvB
PA4304	-7	RcpA
PA4363_iciA	-2.9	inhibitor of chromosome initiation IciA
PA4547_pilR	3.3	two-component response regulator PilR
PA4590_pra	-2.3	protein activator
PA4599_mexC	4.2	RND efflux membrane fusion protein MexC precursor
PA4886	-7	probable two-component sensor
PA5032	10.6	probable transcriptional regulator
PA5059	2.3	probable transcriptional regulator
PA5283	2	probable transcriptional regulator
PA5293	9.7	probable transcriptional regulator
**Motility**		
PA0409_pilH	4.3	twitching motility protein PilH
PA0582_folB	2.2	dihydroneopterin aldolase
PA0993	-5.3	chaperone CupC2
PA1077_flgB	3.2	flagellar basal-body rod protein FlgB
PA1079_flgD	2.5	flagellar basal-body rod modification protein FlgD
PA1080_flgE	2.2	flagellar hook protein FlgE
PA1082_flgG	3.2	flagellar basal-body rod protein FlgG
PA1084_flgI	7	flagellar P-ring protein precursor FlgI
PA1100_fliE	5.9	flagellar hook-basal body complex protein FliE
PA1102_fliG	3.4	flagellar motor switch protein FliG

Gene name	Change fold (ΔBswR/wt)	Gene description
PA1104_fliI	5.7	flagellum-specific ATP synthase FliI
PA1251	2.3	probable chemotaxis transducer
PA1646	2	probable chemotaxis transducer
PA1930	3.9	probable chemotaxis transducer
PA2131	-2.5	fimbrial subunit CupA4
PA2920	-2.7	probable chemotaxis transducer
PA3349_cheR	6.2	probable chemotaxis protein
PA3352_flgN	2.5	hypothetical protein
PA4082	2.3	adhesive protein CupB5
PA4525_pilA	3	type 4 fimbrial precursor PilA
PA4526_pilB	3.3	type 4 fimbrial biogenesis protein PilB
PA4528_pilD	2.4	type 4 prepilin peptidase PilD
PA4549_fimT	5.1	type 4 fimbrial biogenesis protein FimT
PA4550_fimU	3.4	type 4 fimbrial biogenesis protein FimU
PA4551_pilV	4.3	type 4 fimbrial biogenesis protein PilV
PA4552_pilW	3.1	type 4 fimbrial biogenesis protein PilW
PA4554_pilY1	8.4	type 4 fimbrial biogenesis protein PilY1
PA4648	2.7	Pilin subunit CupE1
PA4649	5.6	Pilin subunit CupE2
PA4650	-2	Pilin subunit CupE3
PA4651	-3	Pilin assembly chaperone CupE4
PA4653	-3.5	Adhesin-like protein CupE6
PA4829_lpd3	9.4	dihydrolipoamide dehydrogenase 3
PA5040_pilQ	2	Type 4 fimbrial biogenesis PilQ precursor
PA5042_pilO	2.7	type 4 fimbrial biogenesis protein PilO
PA5043_pilN	2	type 4 fimbrial biogenesis protein PilN
**Secretion**		
PA0044_exoT	2.3	exoenzyme T
PA0071	-2.7	TagR1
PA0072	-2.2	TagS1
PA0075	-2.7	PppA
PA0076	-2.9	TagF1
PA0078	-2.4	TssL1
PA0079	-2.1	TssK1
PA0080	-3.3	TssJ1
PA0081	-2.3	Fha1
PA0082	-3.1	TssA1
PA0083	-4.4	TssB1
PA0084	-3	TssC1
PA0087	-3.5	TssE1
PA0090	-2.2	ClpV1

Gene name	Change fold (ΔBswR/wt)	Gene description
PA0198_exbB1	-3.8	transport protein ExbB
PA0683	-4	probable type II secretion system protein
PA0685	-11.5	probable type II secretion system protein
PA0686	5.6	probable type II secretion system protein
PA0687	3.9	probable type II secretion system protein
PA0693_exbB2	-11.8	transport protein ExbB2
PA1246_aprD	-5.1	alkaline protease secretion protein AprD
PA1691_pscT	2.8	translocation protein in type III secretion
PA1693_pscR	2.2	translocation protein in type III secretion
PA1696_pscO	2.1	translocation protein in type III secretion
PA1697	3.2	ATP synthase in type III secretion system
PA1713_exsA	2.3	transcriptional regulator ExsA
PA1703_pcrD	2.8	type III secretory apparatus protein PcrD
PA1705_pcrG_i	2	regulator in type III secretion
PA1708_popB	5.9	translocator protein PopB
PA1711	2	ExsE
PA1712_exsB	2.5	exoenzyme S synthesis protein B
PA1718_pscE	2.7	type III export protein PscE
PA1719_pscF	3.4	type III export protein PscF
PA1720_pscG	2.2	type III export protein PscG
PA1724_pscK	2.3	type III export protein PscK
PA1868_xqhA	2.8	secretion protein XqhA
PA2672	15.5	probable type II secretion system protein
PA2673	-2.3	probable type II secretion system protein
PA2674	-12.2	probable type II secretion system protein
PA2677	-6.1	probable type II secretion protein
PA2808	5.1	Pseudomonas type III repressor A
PA2836	2.3	probable secretion protein
PA3005_exoS	2.3	beta-N-acetyl-D-glucosaminidase
PA3104_xcpP	-2	secretion protein XcpP
PA3360	23.9	probable secretion protein
PA3405_hasE	-2.1	metalloprotease secretion protein
PA4142	-3.4	probable secretion protein
PA4297	2	TadG
PA4300	8.9	TadC
PA4302	-2.5	TadA ATPase
PA4303	4.1	TadZ
PA4306	-3.1	Type IVb pilin, Flp
**Metabolism**		
PA0796_prpB	2	carboxyphosphonoenolpyruvate phosphonomutase
PA1194	-2.6	probable amino acid permease

Gene name	Change fold (ΔBswR/wt)	Gene description
PA1321_cyoE	2.3	cytochrome o ubiquinol oxidase protein CyoE
PA1384_galE	3	UDP-glucose 4-epimerase
PA1409_aphA	2.4	acetylpolyamine aminohydrolase
PA1410	2	periplasmic spermidine/putrescine-binding protein
PA1525	2.8	alkane-1-monooxygenase 2
PA1600	3.6	probable cytochrome c
PA1905	3.9	probable pyridoxamine 5'-phosphate oxidase
PA1916	-2.1	probable amino acid permease
PA1927_metE	3.7	triglutamate-homocysteine S-methyltransferase
PA2007_maiA	16.7	maleylacetoacetate isomerase
PA2012	-4.1	methylcrotonyl-CoA carboxylase, alpha-subunit
PA2035	-8.3	probable decarboxylase
PA2084	4	probable asparagine synthetase
PA2108	8.7	probable decarboxylase
PA2153_glgB	6.3	1,4-alpha-glucan branching enzyme
PA2202	-2.3	probable amino acid permease
PA2342_mtlD	-3	mannitol dehydrogenase
PA2343_mtlY	2.4	xylulose kinase
PA2357_msuE	-3.3	NADH-dependent FMN reductase MsuE
PA2413	-10.1	L-sorbosone dehydrogenase
PA2442_gcvT2	3.5	L-serine dehydratase
PA2499	5.2	probable deaminase
PA2517_xylY	-8.1	toluate 1,2-dioxygenase beta subunit
PA2518_xylX	8.5	toluate 1,2-dioxygenase alpha subunit
PA2629_purB	2	adenylosuccinate lyase
PA2717_cpo	-2.1	chloroperoxidase precursor
PA2787_cpg2	10.1	carboxypeptidase G2 precursor
PA2862_lipA	2.6	lactonizing lipase precursor
PA2890	3.6	putative isohexenylglutaconyl-CoA hydratase
PA3032	3	cytochrome c Snr1
PA3331	2.5	cytochrome P450
PA3394_nosF	5	NosF protein
PA3395_nosY	-4	NosY protein
PA3396_nosL	6.2	NosL protein
PA3506	8.6	probable decarboxylase
PA3540_algD	5.3	GDP-mannose 6-dehydrogenase AlgD
PA3597	-13.4	probable amino acid permease
PA3871	2.1	peptidyl-prolyl cis-trans isomerase, PpiC-type
PA3874_narH	9.9	respiratory nitrate reductase beta chain
PA3938	-5	periplasmic taurine-binding protein precursor
PA4088	4.1	probable aminotransferase
PA4110_ampC	3.1	beta-lactamase precursor

Gene name	Change fold (ΔBswR/wt)	Gene description
PA4125_hpcD	-2.3	5-carboxymethyl-2-hydroxymuconate isomerase
PA4127_hpcG	2.3	2-oxo-hept-3-ene-1,7-dioate hydratase
PA4151_acoB	2	acetoin catabolism protein AcoB
PA4153	-2.2	2,3-butanediol dehydrogenase
PA4201_ddlA	-2.3	D-alanine-D-alanine ligase A
PA4827	-14	arylamine N-acetyltransferase
PA4901_mdlC	-13.3	benzoylformate decarboxylase
PA4904_vanA	15.5	vanillate O-demethylase oxygenase subunit
PA4905_vanB	-3.3	vanillate O-demethylase oxidoreductase
PA4945_miaA	2.1	delta 2-isopentenylpyrophosphate transferase
PA4976_aspC	4.9	Arginine:Pyruvate Transaminas, AruH
PA5386	2.5	CdhA, Carnitine dehydrogenase
PA5390	7.4	probable peptidic bond hydrolase
PA5394_cls	2.3	cardiolipin synthase
PA5398	3.6	DgcA, Dimethylglycine catabolism
**Adaptation and protection**		
PA0059_osmC	2.5	osmotically inducible protein OsmC
PA0074_ppkA	-2.5	serine/threonine protein kinase PpkA
PA0122	-2	rahU
PA0189	-11.5	probable porin
PA0240	3.6	probable porin
PA0287	3.2	3-guanidinopropionate transport protein
PA0470	2.5	Ferrichrome receptor FiuA
PA0519_nirS	-2.4	nitrite reductase precursor
PA0523_norC	-3.6	nitric-oxide reductase subunit C
PA0524_norB	-2.9	nitric-oxide reductase subunit B
PA0534	-2.3	FAD-dependent oxidoreductase
PA0724	-2	probable coat protein A of bacteriophage Pf1
PA0807	2.7	AmpDh3
PA0880	-2.4	probable ring-cleaving dioxygenase
PA0998	-2.6	PqsC
PA0999_fabH1	4.8	3-oxoacyl-[acyl-carrier-protein] synthase III
PA1000	8.3	Quinolone signal response protein
PA1001_phnA	-2.2	anthranilate synthase component I
PA1025	5.5	probable porin
PA1130	2	rhamnosyltransferase 2
PA1174_napA	-3.4	periplasmic nitrate reductase protein NapA
PA1176_napF	-2.6	ferredoxin protein NapF
PA1253	12.6	probable semialdehyde dehydrogenase
PA1505_moaA2	-2.3	molybdopterin biosynthetic protein A2
PA1549	2	probable cation-transporting P-type ATPase
PA1565	-11	FAD-dependent oxidoreductase

Gene name	Change fold (ΔBswR/wt)	Gene description
PA1632_kdpF	4	KdpF protein
PA1670_stp1	7.5	serine/threonine phosphoprotein phosphatase Stp1
PA1700	2.3	Pcr2
PA1871_lasA	3.1	LasA protease precursor
PA1902	-3.4	phenazine biosynthesis protein PhzD
PA1903	2.3	phenazine biosynthesis protein PhzE
PA1904	5.1	probable phenazine biosynthesis protein
PA1919	10.9	ribonucleoside-triphosphate reductase, 'activase', NrdG
PA1976	16.4	ErcS'
PA2147_katE	12	catalase HPII
PA2185	4.1	non-heme catalase KatN
PA2241	2.1	PslL
PA2254_pvcA	-3.1	paerucumarin biosynthesis protein PvcA
PA2255_pvcB	-7.2	paerucumarin biosynthesis protein PvcB
PA2257_pvcD	4.1	paerucumarin biosynthesis protein PvcD
PA2356_msuD	12.5	methanesulfonate sulfonatase MsuD
PA2385	2.7	3-oxo-C12-homoserine lactone acylase PvdQ
PA2394	-10	PvdN
PA2395	-3.5	PvdO
PA2396	3	pyoverdine synthetase F
PA2397_pvdE	2.1	pyoverdine biosynthesis protein PvdE
PA2398_fpvA	4.4	ferripyoverdine receptor
PA2399_pvdD	2	pyoverdine synthetase D
PA2400	-9.1	PvdJ
PA2423	-5.5	PvdL
PA2424	-5.2	PvdG
PA2512_antA	2.4	anthranilate dioxygenase large subunit
PA2513_antB	-2	anthranilate dioxygenase small subunit
PA2664_fhp	-3.1	flavohemoprotein
PA2700	2.9	proline porin OpdB
PA2749_endA	2.2	DNA-specific endonuclease I
PA2887	-15.5	putative dehydrogenase involved in citronellol catabolism
PA2893	-4.6	putative very-long chain acyl-CoA synthetase
PA2923_hisJ	3	periplasmic histidine-binding protein HisJ
PA3024	4.9	probable carbohydrate kinase
PA3058-pelG	-2.9	PelG
PA3059-pelF	-2.3	PelF
PA3061-pelD	-2.4	PelD
PA3063-pelB	-2.8	PelB
PA3064-pelA	-4.7	PelA
PA3382_phnE	-2	phosphonate transport protein PhnE
PA3392_nosZ	-2.2	nitrous-oxide reductase precursor
PA3478_rhlB	-2	rhamnosyltransferase chain B
PA3479_rhlA	-6.2	rhamnosyltransferase chain A
PA3548_algI	3.1	alginate o-acetyltransferase AlgI

Gene name	Change fold (ΔBswR/wt)	Gene description
PA3549_algJ	6.8	alginate o-acetyltransferase AlgJ
PA3550_algF	-3.3	alginate o-acetyltransferase AlgF
PA3588	3.1	probable porin
PA3692	2.6	Lipotoxon F, LptF
PA3724_lasB	2.3	elastase LasB
PA3870_moaA1	10.8	molybdopterin biosynthetic protein A1
PA3877_narK1	-2.3	nitrite extrusion protein 1
PA3909	-14.7	Extracelullar DNA degradation protein, EddB
PA4034_aqpZ	-8	aquaporin Z
PA4091_hpaA	16.2	4-hydroxyphenylacetate 3-monooxygenase large chain
PA4092_hpaC	-4.7	4-hydroxyphenylacetate 3-monooxygenase small chain
PA4137	-5.3	probable porin
PA4175	-2.3	protease IV
PA4209	2.6	probable phenazine-specific methyltransferase
PA4211	-7.7	probable phenazine biosynthesis protein
PA4217	2.4	flavin-containing monooxygenase
PA4218	2	AmpP
PA4228_pchD	2.5	pyochelin biosynthesis protein PchD
PA4231_pchA	2	salicylate biosynthesis isochorismate synthase
PA4283_recD	-2.1	exodeoxyribonuclease V alpha chain
PA4295	-2.4	Flp prepilin peptidase A, FppA
PA4339	-2	probable phospholipase
PA4624	3.4	cyclic diguanylate-regulated TPS partner B, CdrB
PA4625	2.4	cyclic diguanylate-regulated TPS partner A, CdrA
PA4781	-3.8	cyclic di-GMP phosphodiesterase
PA5127	2.2	probable rRNA methylase
PA5416_soxB	-17.3	sarcosine oxidase beta subunit
PA5418_soxA	21.1	sarcosine oxidase alpha subunit
PA5536	2.5	DksA2
**Transporter and membrane proteins**		
PA0137	5.1	probable permease of ABC transporter
PA0138	-3.5	probable permease of ABC transporter
PA0157	2.1	RND triclosan efflux membrane fusion protein, TriB
PA0184	5.8	probable ATP-binding component of ABC transporter
PA0204	39.5	probable permease of ABC transporter
PA0206	-3.6	probable ATP-binding component of ABC transporter
PA0280_cysA	-4	sulfate transport protein CysA
PA0465_creD	-5.5	inner membrane protein CreD
PA0886	-2	probable C4-dicarboxylate transporter
PA1019_mucK	-5.4	cis,cis-muconate transporter MucK
PA1260	5.2	amino acid ABC transporter periplasmic binding protein
PA1496	-2.5	probable potassium channel
PA1635_kdpC	2.6	potassium-transporting ATPase, C chain

Gene name	Change fold (ΔBswR/wt)	Gene description
PA1783_nasA	-2.3	nitrate transporter
PA2018	-10.5	RND multidrug efflux transporter
PA2302	4.2	AmbE
PA2494_mexF	-2.3	RND multidrug efflux transporter MexF
PA2521_czcB	-8.7	RND divalent metal cation efflux protein CzcB precursor
PA2527	2	probable RND efflux transporter
PA3383	-10.6	ABC phosphonate transporter
PA3384_phnC	4.4	ABC phosphonate transporter
PA3522	11	probable RND efflux transporter
PA3523	6	probable RND efflux membrane protein precursor
PA3671	11	probable permease of ABC transporter
PA3888	-4.8	probable permease of ABC transporter
PA4160_fepD	-4.3	ferric enterobactin transport protein FepD
PA4207	2.2	probable RND efflux transporter
PA4374	-2.3	RND efflux membrane fusion protein precursor
PA4504	-3.3	probable permease of ABC transporter
PA4593	-5.2	probable permease of ABC transporter
PA4594	8.8	probable ATP-binding component of ABC transporter
PA4859	2	probable permease of ABC transporter
PA4860	5.7	probable permease of ABC transporter
PA4910	-7.4	ABC transporter ATP binding protein
PA4911	3.3	ABC branched-chain amino acid transporter
PA4912	-4.1	ABC transporter membrane protein
PA5207	2.2	probable phosphate transporter
PA5216	-3.6	probable permease of ABC iron transporter
PA5287_amtB	3.3	ammonium transporter AmtB
PA5468	2.1	probable citrate transporter
**Hypothetic proteins**		
PA0718	-2.8	hypothetical protein of bacteriophage Pf1
PA0721	-4.4	hypothetical protein of bacteriophage Pf1
PA0725	2	hypothetical protein of bacteriophage Pf1
PA3358	-5.6	hypothetical protein
PA3359	2.2	hypothetical protein
PA3422	6	hypothetical protein
PA3464	2.1	hypothetical protein
PA3501	2.5	hypothetical protein
PA3789	-3.7	hypothetical protein
PA3825	-5.5	hypothetical protein
PA3840	2.9	conserved hypothetical protein
PA3847	-2.3	conserved hypothetical protein
PA3855	-2.3	hypothetical protein
PA3897	-2.4	hypothetical protein

Gene name	Change fold (ΔBswR/wt)	Gene description
PA3908	4.8	hypothetical protein
PA3928	10.3	hypothetical protein
PA3931	-2	conserved hypothetical protein
PA3939	-2.3	hypothetical protein
PA3944	-2.1	conserved hypothetical protein
PA3954	-6.1	hypothetical protein
PA3964	5.2	hypothetical protein
PA4028	-2.5	hypothetical protein
PA4038	4.2	hypothetical protein
PA4062	-2.1	hypothetical protein
PA4065	2.1	hypothetical protein
PA4071	2.9	hypothetical protein
PA4075	-8.6	hypothetical protein
PA4087	-6.8	conserved hypothetical protein
PA4093	-5.1	hypothetical protein
PA4103	7.2	hypothetical protein
PA4106	4.6	conserved hypothetical protein
PA4107	2	hypothetical protein
PA4121	-3.1	conserved hypothetical protein
PA4128	-7	conserved hypothetical protein
PA4134	2	hypothetical protein
PA4140	9	hypothetical protein
PA4173	-2.7	conserved hypothetical protein
PA4186	2.3	hypothetical protein
PA4188	4.7	conserved hypothetical protein
PA4205	-2.5	hypothetical protein
PA4287	-2.7	hypothetical protein
PA4323	-3	hypothetical protein
PA4335	-8.9	hypothetical protein
PA4337	2.3	hypothetical protein
PA4345	2.1	hypothetical protein
PA4362	-9.5	hypothetical protein
PA4467	-10.1	hypothetical protein
PA4474	2.1	conserved hypothetical protein
PA4485	4	conserved hypothetical protein
PA4509	2.3	hypothetical protein
PA4510	2	conserved hypothetical protein
PA4540	5.9	hypothetical protein
PA4541	-3.1	hypothetical protein
PA4584	-5.8	conserved hypothetical protein
PA4586	25.1	hypothetical protein
PA4617	-2.2	conserved hypothetical protein
PA4629	2.1	hypothetical protein
PA4634	-5.1	hypothetical protein
PA4635	5	conserved hypothetical protein

Gene name	Change fold (ΔBswR/wt)	Gene description
PA4642	-3.5	hypothetical protein
PA4677	-4.4	hypothetical protein
PA4680	-2.9	hypothetical protein
PA4685	3.1	hypothetical protein
PA4713	2.5	hypothetical protein
PA4779	2.1	hypothetical protein
PA4788	-7.3	hypothetical protein
PA4828	-2.1	conserved hypothetical protein
PA4858	2.4	conserved hypothetical protein
PA4926	2	conserved hypothetical protein
PA5101	18.8	hypothetical protein
PA5102	-2.5	hypothetical protein
PA5103	-3.1	hypothetical protein
PA5183	2.3	hypothetical protein
PA5202	2.1	hypothetical protein
PA5227	-2	conserved hypothetical protein
PA5265	5.1	hypothetical protein
PA5294	3.3	hypothetical protein
PA5325	-2.3	hypothetical protein
PA5326	-4.5	hypothetical protein
PA5383	2.9	conserved hypothetical protein
PA5391	2.5	hypothetical protein
PA5392	2.7	conserved hypothetical protein
PA5397	-16.4	hypothetical protein
PA5401	9.2	hypothetical protein
PA5442	2.4	conserved hypothetical protein
PA5455	-2.3	hypothetical protein
PA5456	-2.1	hypothetical protein
PA5532	3.5	hypothetical protein
